# Supplementary material for: Global Healthcare Needs Related to COVID-19: An Evidence Map of the First Year of the Pandemic
Source: Int J Environ Res Public Health. 2022 Aug 19;19(16):10332. doi: 10.3390/ijerph191610332 (PMC9408445; doi:10.3390/ijerph191610332)
Supplement: Supplementary file 1 [file ijerph-19-10332-s001.zip › S2_SearchStrategy_17-08-2022.pdf]

Table S2. Search strategy used in the consulted databases

Consistent with the nature of the present systematic evidence map, a broad search strategy was used to cover a wider scope of evidence including all publications associated with healthcare related needs during the first year of the COVID-19 pandemic.

Dates: 2019 to January 14, 2021

Databases: MEDLINE, Embase, Web of Science

MEDLINE

Access platform: Ovid SP

Search Strategy:

No.	Query	Results	Annotations
1	(coronavirus or "covid19" or covid-19 or sars* or "2019-ncov" or "covid-2019" or "severe acute respiratory syndrome coronavirus 2").ti,ab,kw.	108159	Concept: ***COVID-19***
2	Health Personnel/	45907	#2 to #5: Concept: ***Healthcare professionals***
3	Health Occupations/	8428	
4	health care worker*.ti,ab.	13998	
5	(health adj2 (personnel or occupation*)).ti,ab.	23191	
6	1 or 2 or 3 or 4 or 5	190303	***COVID-19*** OR ***Healthcare professionals***
7	Needs Assessment/	30900	Concept: ***Needs Assessment***
8	((assessment or determination) adj3 ("health care needs" or "healthcare needs")).mp. [mp=title, abstract, original title, name of substance word, subject heading word, floating sub-heading word, keyword heading word, organism supplementary concept word, protocol supplementary concept word, rare disease supplementary concept word, unique identifier, synonyms]	103	Concept: ***Healthcare needs assessment***
9	((healthcare or social or support) adj need*).ti,ab,kw.	7405	Concept: ***Healthcare or related needs***
10	7 or 8 or 9	37658	***Needs assessment*** OR ***Healthcare needs assessment*** OR ***Healthcare or related needs***

No.	Query	Results	Annotations
11	6 and 10	1442	(***COVID-19*** OR ***Healthcare professionals***) AND (***Needs assessment*** OR ***Healthcare needs assessment*** OR ***Healthcare or related needs***)
12	limit 11 to yr="2019 -Current"	**452**	(***COVID-19*** OR ***Healthcare professionals***) AND (***Needs assessment*** OR ***Healthcare needs assessment*** OR ***Healthcare or related needs***) limited by dates specified (limits results to *2019 – current*). **Final result**

Embase

Access platform: Elsevier

Search strategy:

No.	Query	Results	Annotations
12	#6 AND #10 AND [2019-2021]/py	541	(***COVID-19*** OR ***Healthcare professionals***) AND (***Needs assessment*** OR ***Healthcare needs assessment*** OR ***Healthcare or related needs***) limited by dates specified (limits results to *2019 – 2021*). **Final result**
11	#6 AND #10	12,272	(***COVID-19*** OR ***Healthcare professionals***) AND (***Needs assessment*** OR ***Healthcare needs assessment*** OR ***Healthcare or related needs***)
10	#7 OR #8 OR #9	37,138	***Needs assessment*** OR ***Healthcare needs assessment*** OR ***Healthcare or related needs***
9	((healthcare OR social OR support) NEAR/1 need*):ti,ab,de	13,006	Concept: ***Healthcare or related needs***

No.	Query	Results	Annotations
8	((assessment OR determination) NEAR/3 ('health care needs' OR 'healthcare needs')):ti,ab,de	125	Concept: ***Healthcare needs assessment***
7	'needs assessment'/exp	24,799	Concept: ***Needs Assessment***
6	#1 OR #2 OR #3 OR #4 OR #5	342,452	***COVID-19*** OR ***Healthcare professionals***
5	((health OR medical) NEAR/2 (personnel OR occupation* OR profession*)):ti,ab	174,155	#2 to #5: Concept: ***Healthcare professionals***
4	'health care worker*':ti,ab	16,922	
3	'medical profession'/exp	27,247	
2	'health care personnel'/mj	29,968	
1	coronavirus:ti,ab,de OR 'covid19':ti,ab,de OR 'covid 19':ti,ab,de OR sars*:ti,ab,de OR '2019-ncov':ti,ab,de OR 'covid-2019':ti,ab,de OR 'severe acute respiratory syndrome coronavirus 2':ti,ab,de	113,811	Concept: ***COVID-19***

Web of Science

Access platform: Clarivate

Search strategy:

No.	Query	Results	Annotations
9	#8 limited to *2019 – 2021*	633	(***Healthcare or related needs*** OR ***Healthcare needs assessment***) AND (***COVID-19*** OR ***Healthcare professionals***) limited by dates specified (limits results to *2019 – 2021*). **Final result**
8	#7 AND #4	1,314	(***Healthcare or related needs*** OR ***Healthcare needs assessment***) AND

No.	Query	Results	Annotations
			(***COVID-19*** OR ***Healthcare professionals***)
7	#6 OR #5	46,948	***Healthcare or related needs*** OR ***Healthcare needs assessment***
6	TS= ((healthcare or social or support) NEAR/1 need*)	46,861	Concept: ***Healthcare or related needs***
5	TS= ((assessment or determination) NEAR/3 ("health care needs" or "healthcare needs"))	12	Concept: ***Healthcare needs assessment***
4	#3 OR #2 OR #1	174,049	***COVID-19*** OR ***Healthcare professionals***
3	TS= (coronavirus or "covid19" or covid-19 or sars* or "2019-ncov" or "covid-2019" or "severe acute respiratory syndrome coronavirus 2")	102,959	Concept: ***COVID-19***
2	TS= health care worker*	42,186	#1 to #2: Concept: ***Healthcare professionals***
1	TS= (health NEAR/2 (personnel or occupation*))	34,687	
