# Supplementary material for: Global Healthcare Needs Related to COVID-19: An Evidence Map of the First Year of the Pandemic
Source: Int J Environ Res Public Health. 2022 Aug 19;19(16):10332. doi: 10.3390/ijerph191610332 (PMC9408445; doi:10.3390/ijerph191610332)
Supplement: Supplementary file 1 [file ijerph-19-10332-s001.zip › S3_ExcludedStudies_17-08.2022.pdf]

Table S3. List of excluded studies and reasons for exclusion

Reports not retrieved, but sought for retrieval (i.e., No full text available; n = 2)

Full-text articles excluded, with reasons (n = 107)

Ineligible population (n =10)
Ineligible outcome (n =32)
Non-primary work (n =65)

**First Author**	**Year**	**Reason for exclusion**
Ahojes, L. [1]	2020	No full-text available
Albutt, K.[2]	2020	Ineligible outcome
Almater, A.I.[3]	2020	Ineligible outcome
Ayalon, L. [4]	2020	Non-primary work
Ayalon, L. [5]	2020	Non-primary work
Barker, J. [6]	2021	Non-primary work
Barratt, R. [7]	2019	Non-primary work
Barth, R.F. [8]	2020	Non-primary work
Belli, S. [9]	2020	Ineligible outcome
Berkowitz, S.A. [10]	2021	Ineligible population
Bhatia, A. [11]	2020	No full-text available
Bickerton, L. [12]	2020	Ineligible population
Bielicki, J.A. [13]	2020	Non-primary work
Boldrini, P.[14]	2020	Ineligible outcome
Bressy, S. [15]	2020	Ineligible outcome
Brennan, J. [16]	2020	Non-primary work
Butler, M. [17]	2020	Non-primary work
Cable, N. [18]	2020	Non-primary work

**First Author**	**Year**	**Reason for exclusion**
Caesar, S. [19]	2020	Non-primary work
Choo, E,K. [20]	2020	Non-primary work
Clapp, J. [21]	2020	Non-primary work
Cortese, B. [22]	2020	Non-primary work
Davuluri, M. [23]	2020	Ineligible outcome
Deep, A. [24]	2021	Ineligible outcome
De la Rosette, J. [25]	2020	Ineligible outcome
Del Buono, M.G.[26]	2020	Non-primary work
De Leo, D.[27]	2020	Non-primary work
Dramowski, A. [28]	2020	Ineligible outcome
El-Awaisi, A. [29]	2020	Ineligible outcome
Fisher, B. [30]	2020	Ineligible outcome
Fish, J.N. [31]	2021	Ineligible population
Ford, C.L. [32]	2020	Non-primary work
Freeman, J. [33]	2020	Non-primary work
Freischlag, J.A.[34]	2020	Non-primary work
Gaffney, A.W. [35]	2020	Ineligible outcome
Glasby, J. [36]	2020	Non-primary work
Goertz, Y.M.J. [37]	2020	Ineligible outcome
Goldfarb, D.S. [38]	2020	Non-primary work
Gondi, S. [39]	2020	Non-primary work
Gray, D.M. [40]	2020	Non-primary work
Halcomb, L.[41]	2020	Ineligible population

First Author	Year	Reason for exclusion
Hansmann, K.J. [42]	2020	Non-primary work
Harris, D.A. [43]	2021	Ineligible outcome
Hategan, A. [44]	2020	Non-primary work
Hebbar, P.B. [45]	2020	Non-primary work
Hofmeyer, A. [46]	2021	Non-primary work
Hwang, T.J.[47]	2020	Non-primary work
Iacobucci, G. [48]	2020	Non-primary work
Iyengar, K. [49]	2020	Non-primary work
Jackson, C.L. [50]	2020	Non-primary work
Kar, S.K. [51]	2020	Non-primary work
Khoury, R. [52]	2020	Non-primary work
Kim, E.J. [53]	2020	Non-primary work
Koffman, J. [54]	2020	Non-primary work
Krist, A.H. [55]	2020	Non-primary work
Krumholz, H.M. [56]	2020	Ineligible outcome
Lam, K. [57]	2020	Ineligible outcome
Lee, E.[58]	2020	Ineligible outcome
Mahase, E. [59]	2020	Non-primary work
Majeed, A. [60]	2020	Non-primary work
Markowitz, J. [61]	2020	Non-primary work
Mateos, R.[62]	2020	Non-primary work
Maulik, P.K. [63]	2020	Non-primary work
McGarry, B.E. [64]	2020	Ineligible outcome

First Author	Year	Reason for exclusion
Meagher, K.M. [65]	2020	Non-primary work
Medina-Walpole, A. [66]	2020	Non-primary work
Mein, S.A. [67]	2020	Non-primary work
Mills, K.T. [68]	2021	Ineligible outcome
Miller, A. [69]	2020	Non-primary work
Monjur, M.R. [70]	2020	Non-primary work
Moynihan, R. [71]	2020	Non-primary work
Naser, A.Y. [72]	2020	Ineligible outcome
Naharci, M.I. [73]	2020	Non-primary work
O'Connor, D. [74]	2020	Non-primary work
O'Reilly-Shan, V.N. [75]	2020	Ineligible outcome
Page, N. [76]	2020	Ineligible population
Pai, R.R. [77]	2020	Ineligible outcome: This is an example of a study that addressed needs in the general population, but not specifically in our population subgroups.
Poussardin, C. [78]	2020	Ineligible outcome
Prior, M. [79]	2020	Ineligible outcome
Rascado Sedes, P. [80]	n.d	Ineligible outcome
Raffle, A.E. [81]	2020	Non-primary work
Rana, U. [82]	2020	Non-primary work
Ray, K.N. [83]	2021	Ineligible population
Reger, M.A. [84]	2020	Non-primary work
Rimmer, A. [85]	2020	Ineligible outcome

First Author	Year	Reason for exclusion
Rokach, A. [86]	2020	Ineligible population
Sandhu, S. [87]	2021	Ineligible outcome
Schlesinger, T. [88]	2020	Non-primary work
Scott, I.A. [89]	2020	Non-primary work
Sebo, P. [90]	2020	Ineligible outcome
Siegelman, J.N. [91]	2020	Non-primary work
Sienaert, P. [92]	2020	Non-primary work
Sivashanker, K. [93]	n.d.	Non-primary work
Skoog, I. [94]	2020	Non-primary work
Spooner, A. [95]	2020	Ineligible outcome
Stephenson, K. [96]	2020	Ineligible outcome
Suzuki, M. [97]	2020	Ineligible population
Swain, K. [98]	2020	Non-primary work
Vedavanam, K. [99]	2020	Ineligible population
Veitch, P. [100]	2021	Non-primary work
Wand, A.P.F. [101]	2020	Non-primary work
Wang, Z. [102]	2021	Ineligible outcome
Watterson, A. [103]	2020	Non-primary work
Weiss, P.G. [104]	2020	Non-primary work
Wilson, A.N. [105]	2021	Non-primary work
Xiang, Y.T. [106]	2020	Non-primary work
Xie, H.L. [107]	2020	Ineligible outcome: This is an example of a study that focused on exploring the prevalence of a mental health illness or psychological factor, but did not aim to identify healthcare or

**First Author**	**Year**	**Reason for exclusion**
		related needs associated with COVID-19 from the perspective of one of the population subgroups.
Zeenny, R.M. [108]	2020	Ineligible population
Fundery Efforts [109]	2020	Non-primary work

References

1. Ahojes, L. CN33 Level and Stress Factors among Nursing Professionals of the Units with Onco-Haematological Patients Diagnosed of COVID-19. *Annals of Oncology* **2020**, *31*, S1138–S1139, doi:10.1016/j.annonc.2020.08.2141.
2. Albutt, K.; Luckhurst, C.M.; Alba, G.A.; Hechi, M.E.; Mokhtari, A.; Breen, K.; Wing, J.; Akeju, O.; Kalva, S.P.; Mullen, J.T.; et al. Design and Impact of a COVID-19 Multidisciplinary Bundled Procedure Team. *Annals of Surgery* **2020**, *272*, e72–e73, doi:10.1097/SLA.0000000000004089.
3. Almater, A.; Tobaigy, M.; Younis, A.; Alaqeel, M.; Abouammoh, M. Effect of 2019 Coronavirus Pandemic on Ophthalmologists Practicing in Saudi Arabia: A Psychological Health Assessment. *Middle East Afr J Ophthalmol* **2020**, *27*, 79–85, doi:10.4103/meajo.MEAJO_220_20.
4. Ayalon, L. There Is Nothing New under the Sun: Ageism and Intergenerational Tension in the Age of the COVID-19 Outbreak. *Int. Psychogeriatr.* **2020**, *32*, 1221–1224, doi:10.1017/S1041610220000575.
5. Ayalon, L.; Zisberg, A.; Cohn-Schwartz, E.; Cohen-Mansfield, J.; Perel-Levin, S.; Bar-Asher Siegal, E. Long-Term Care Settings in the Times of COVID-19: Challenges and Future Directions. *Int. Psychogeriatr.* **2020**, *32*, 1239–1243, doi:10.1017/S1041610220001416.
6. Baker, K.E.; Wilson, L.M.; Sharma, R.; Dukhanin, V.; McArthur, K.; Robinson, K.A. Hormone Therapy, Mental Health, and Quality of Life Among Transgender People: A Systematic Review. *Journal of the Endocrine Society* **2021**, *5*, bvab011, doi:10.1210/jendso/bvab011.
7. Barratt, R.; Shaban, R.Z.; Gilbert, G.L. Clinician Perceptions of Respiratory Infection Risk; a Rationale for Research into Mask Use in Routine Practice. *Infection, Disease & Health* **2019**, *24*, 169–176, doi:10.1016/j.idh.2019.01.003.
8. Barth, R.F.; Xu, X.; Buja, L.M. A Call to Action. *Chest* **2020**, *158*, 43–44, doi:10.1016/j.chest.2020.03.060.
9. Belli, S.; Balbi, B.; Prince, I.; Cattaneo, D.; Masocco, F.; Zaccaria, S.; Bertalli, L.; Cattini, F.; Lomazzo, A.; Dal Negro, F.; et al. Low Physical Functioning and Impaired Performance of Activities of Daily Life in COVID-19 Patients Who Survived Hospitalisation. *Eur Respir J* **2020**, *56*, 1–4, doi:10.1183/13993003.02096-2020.
10. Berkowitz, S.A.; Basu, S. Unemployment Insurance, Health-Related Social Needs, Health Care Access, and Mental Health During the COVID-19 Pandemic. *JAMA Intern Med* **2021**, *181*, 699-702, doi:10.1001/jamainternmed.2020.7048.
11. Bhatia, A.; Waldman, L.; Garber, G.; Sabonjian, M.; Hunt, M.; Palidora, J.; Leader, A.; Worster, B. SKCC Medical Oncology Telehealthexperience during Covid-19 Pandemic: Lessons Learnedand Operational Changes to Leverage Telehealth Tomaintain Patient Access. *Clinical Cancer Research* **2020**.
12. Bickerton, L.; Siegart, N.; Marquez, C. Medical Students Screen for Social Determinants of Health: A Service Learning Model to Improve Health Equity. *PRiMER* **2020**, *4*, 27, doi:10.22454/PRiMER.2020.225894.
13. Bielicki, J.A.; Duval, X.; Gobat, N.; Goossens, H.; Koopmans, M.; Tacconelli, E.; van der Werf, S. Monitoring Approaches for Health-Care Workers during the COVID-19 Pandemic. *The Lancet Infectious Diseases* **2020**, *20*, e261–e267, doi:10.1016/S1473-3099(20)30458-8.
14. Boldrini, P.; Kiekens, C.; Bargellesi, S.; Brianti, R.; Galeri, S.; Lucca, L.; Montis, A.; Posteraro, F.; Scarponi, F.; Straudi, S.; et al. First Impact of COVID-19 on Services and Their Preparation. "Instant Paper from the Field" on Rehabilitation Answers to the COVID-19 Emergency. *Eur J Phys Rehabil Med* **2020**, *56*, doi:10.23736/S1973-9087.20.06303-0.
15. Bressy, S.; Zingarelli, E.M. Technological Devices in COVID-19 Primary Care Management: The Italian Experience. *Family Practice* **2020**, *37*, 725–726, doi:10.1093/fampra/cmaa055.
16. Brennan, J.; Reilly, P.; Cuskelly, K.; Donnelly, S. Social Work, Mental Health, Older People and COVID-19. *Int. Psychogeriatr.* **2020**, *32*, 1205–1209, doi:10.1017/S1041610220000873.
17. Butler, M.; Pollak, T.A.; Rooney, A.G.; Michael, B.D.; Nicholson, T.R. Neuropsychiatric

Complications of Covid-19. *BMJ* **2020**, m3871, doi:10.1136/bmj.m3871.
18. Cable, N. COVID-19 Pandemic: Urgent Needs to Support and Monitor Long-Term Effects of Mental Strain on People. *Am J Public Health* **2020**, *110*, 1595–1596, doi:10.2105/AJPH.2020.305938.
19. Caesar, S.; Layer, G.; Alexander, R.; Banks, V.; Barasi, S.; Bews, S.; Chaudhury, B.; Cockman, P.; Conlon, M.; Dasan, S.; et al. Appraisal Needs to Re-Start Now so Doctors Can Reflect on Coronavirus Experiences. *BMJ* **2020**, m3987, doi:10.1136/bmj.m3987.
20. Choo, E.K.; Rajkumar, S.V. Medication Shortages During the COVID-19 Crisis. *Mayo Clinic Proceedings* **2020**, *95*, 1112–1115, doi:10.1016/j.mayocp.2020.04.001.
21. Clapp, J.; Calvo-Friedman, A.; Cameron, S.; Kramer, N.; Kumar, S.L.; Foote, E.; Lupi, J.; Osuntuyi, O.; Chokshi, D.A. The COVID-19 Shadow Pandemic: Meeting Social Needs For A City In Lockdown: Commentary Describes How New York City Health + Hospitals Staff Developed and Executed a Strategy to Meet Patients' Intensified Social Needs during the COVID-19 Pandemic. *Health Affairs* **2020**, *39*, 1592–1596, doi:10.1377/hlthaff.2020.00928.
22. Cortese, B. COVID-19 Pandemic—Some Cardiovascular Considerations from the Trench. *American Heart Journal* **2020**, *225*, 1–2, doi:10.1016/j.ahj.2020.04.026.
23. Davuluri, M. Urology Chief Resident Turned Medicine Intern: Experience during the COVID-19 New York City Pandemic. *Journal of Urology* **2020**, *204*, 638–639, doi:10.1097/JU.0000000000001137.
24. Deep, A.; Knight, P.; Kernie, S.G.; D'Silva, P.; Sobin, B.; Best, T.; Zorrilla, M.; Carson, L.; Zoica, B.; Ahn, D. A Hybrid Model of Pediatric and Adult Critical Care During the Coronavirus Disease 2019 Surge: The Experience of Two Tertiary Hospitals in London and New York. *Pediatric Critical Care Medicine* **2021**, *22*, e125–e134, doi:10.1097/PCC.0000000000002584.
25. Rosette, J.; Laguna, P.; Álvarez-Maestro, M.; Eto, M.; Mochtar, C.A.; Albayrak, S.; Mendoza-Valdes, A.; Ong, T.A.; Khadgi, S.; Al-Terki, A.; et al. Cross-continental Comparison of Safety and Protection Measures amongst Urologists during COVID-19. *Int. J. Urol.* **2020**, *27*, 981–989, doi:10.1111/iju.14340.
26. Del Buono, M.G.; Iannaccone, G.; Camilli, M.; Del Buono, R.; Aspromonte, N. The Italian Outbreak of COVID-19: Conditions, Contributors, and Concerns. *Mayo Clinic Proceedings* **2020**, *95*, 1116–1118, doi:10.1016/j.mayocp.2020.04.003.
27. De Leo, D.; Trabucchi, M. The Fight against COVID-19: A Report from the Italian Trenches. *Int. Psychogeriatr.* **2020**, *32*, 1161–1164, doi:10.1017/S1041610220000630.
28. Dramowski, A.; Zunza, M.; Dube, K.; Parker, M.; Slogrove, A. South African Healthcare Workers and COVID-19: A Shared Responsibility to Protect a Precious and Limited Resource. *S Afr Med J* **2020**, *110*, 0, doi:10.7196/SAMJ.2020.v110i7.14903.
29. El-Awaisi, A.; O'Carroll, V.; Koraysh, S.; Koummich, S.; Huber, M. Perceptions of Who Is in the Healthcare Team? A Content Analysis of Social Media Posts during COVID-19 Pandemic. *Journal of Interprofessional Care* **2020**, *34*, 622–632, doi:10.1080/13561820.2020.1819779.
30. Fisher, B.; Seese, L.; Sultan, I.; Kilic, A. The Importance of Repeat Testing in Detecting Coronavirus Disease 2019 (COVID-19) in a Coronary Artery Bypass Grafting Patient. *J Card Surg* **2020**, *35*, 1342–1344, doi:10.1111/jocs.14604.
31. Fish, J.N.; Mittal, M. Mental Health Providers During COVID-19: Essential to the US Public Health Workforce and in Need of Support. *Public Health Rep* **2021**, *136*, 14–17, doi:10.1177/0033354920965266.
32. Ford, C.L. Commentary: Addressing Inequities in the Era of COVID-19: The Pandemic and the Urgent Need for Critical Race Theory. *Family & Community Health* **2020**, *43*, 184–186, doi:10.1097/FCH.0000000000000266.
33. Freeman, J. Something Old, Something New: The Syndemic of Racism and COVID-19 and Its Implications for Medical Education. *Fam Med* **2020**, *52*, 623–625, doi:10.22454/FamMed.2020.140670.
34. Freischlag, J.A. Patient Safety Indicators: Inpatient, Outpatient, and COVID 19. *Annals of Surgery* **2020**, *272*, 620–620, doi:10.1097/SLA.0000000000004192.
35. Gaffney, A.W.; Hawks, L.; Bor, D.H.; Woolhandler, S.; Himmelstein, D.U.; McCormick, D. 18.2

Million Individuals at Increased Risk of Severe COVID-19 Illness Are Un- or Underinsured. *J GEN INTERN MED* **2020**, *35*, 2487–2489, doi:10.1007/s11606-020-05899-8.
36. Glasby, J.; Needham, C. The Neglect of Adult Social Care during Covid-19. *BMJ* **2020**, m3103, doi:10.1136/bmj.m3103.
37. Goërtz, Y.M.J.; Van Herck, M.; Delbressine, J.M.; Vaes, A.W.; Meys, R.; Machado, F.V.C.; Houben-Wilke, S.; Burtin, C.; Posthuma, R.; Franssen, F.M.E.; et al. Persistent Symptoms 3 Months after a SARS-CoV-2 Infection: The Post-COVID-19 Syndrome? *ERJ Open Res* **2020**, *6*, 00542–02020, doi:10.1183/23120541.00542-2020.
38. Goldfarb, D.S.; Benstein, J.A.; Zhdanova, O.; Hammer, E.; Block, C.A.; Caplin, N.J.; Thompson, N.; Charytan, D.M. Impending Shortages of Kidney Replacement Therapy for COVID-19 Patients. *CJASN* **2020**, *15*, 880–882, doi:10.2215/CJN.05180420.
39. Gondi, S.; Beckman, A.L.; Deveau, N.; Raja, A.S.; Ranney, M.L.; Popkin, R.; He, S. Personal Protective Equipment Needs in the USA during the COVID-19 Pandemic. *The Lancet* **2020**, *395*, e90–e91, doi:10.1016/S0140-6736(20)31038-2.
40. Gray, D.M.; Anyane-Yeboa, A.; Balzora, S.; Issaka, R.B.; May, F.P. COVID-19 and the Other Pandemic: Populations Made Vulnerable by Systemic Inequity. *Nat Rev Gastroenterol Hepatol* **2020**, *17*, 520–522, doi:10.1038/s41575-020-0330-8.
41. Halcomb, E.; Williams, A.; Ashley, C.; McInnes, S.; Stephen, C.; Calma, K.; James, S. The Support Needs of Australian Primary Health Care Nurses during the COVID-19 Pandemic. *Journal of Nursing Management* **2020**, *28*, 1553–1560, doi:10.1111/jonm.13108.
42. Hansmann, K.J.; Kind, A.J.H. Community Health Workers And COVID-19. *Health Affairs* **2020**, *39*, 1097–1097, doi:10.1377/hlthaff.2020.00554.
43. Harris, D.A.; Archbald-Pannone, L.; Kaur, J.; Cattell-Gordon, D.; Rheuban, K.S.; Ombres, R.L.; Albero, K.; Steele, R.; Bell, T.D.; Mutter, J.B. Rapid Telehealth-Centered Response to COVID-19 Outbreaks in Postacute and Long-Term Care Facilities. *Telemedicine and e-Health* **2021**, *27*, 102–106, doi:10.1089/tmj.2020.0236.
44. Hategan, A.; Abdurrahman, M. Hidden in Plain Sight: Addressing the Unique Needs of High-risk Psychiatric Populations during the COVID -19 Pandemic. *Psychiatry Clin. Neurosci.* **2020**, *74*, 439–439, doi:10.1111/pcn.13022.
45. Hebbar, P.B.; Sudha, A.; Dsouza, V.; Chilgod, L.; Amin, A. Healthcare Delivery in India amid the Covid-19 Pandemic: Challenges and Opportunities. *IJME* **2020**, *05*, 215–218, doi:10.20529/IJME.2020.064.
46. Hofmeyer, A.; Taylor, R. Strategies and Resources for Nurse Leaders to Use to Lead with Empathy and Prudence so They Understand and Address Sources of Anxiety among Nurses Practising in the Era of COVID-19. *J Clin Nurs* **2021**, *30*, 298–305, doi:10.1111/jocn.15520.
47. Hwang, T.-J.; Rabheru, K.; Peisah, C.; Reichman, W.; Ikeda, M. Loneliness and Social Isolation during the COVID-19 Pandemic. *Int. Psychogeriatr.* **2020**, *32*, 1217–1220, doi:10.1017/S1041610220000988.
48. Iacobucci, G. Covid-19: GPs Need Extra Support to Withstand Second Wave, BMA Warns. *BMJ* **2020**, m3806, doi:10.1136/bmj.m3806.
49. Iyengar, K.; Bahl, S.; Raju Vaishya; Vaish, A. Challenges and Solutions in Meeting up the Urgent Requirement of Ventilators for COVID-19 Patients. *Diabetes & Metabolic Syndrome: Clinical Research & Reviews* **2020**, *14*, 499–501, doi:10.1016/j.dsx.2020.04.048.
50. Jackson, C.L.; Johnson, D.A. Sleep Disparities in the Era of the COVID-19 Pandemic Highlight the Urgent Need to Address Social Determinants of Health like the Virus of Racism. *Journal of Clinical Sleep Medicine* **2020**, *16*, 1401–1402, doi:10.5664/jcsm.8570.
51. Kar, S.K.; Singh, N. Person-Centered Approach to the Diverse Mental Healthcare Needs During COVID 19 Pandemic. *SN Compr. Clin. Med.* **2020**, *2*, 1358–1360, doi:10.1007/s42399-020-00428-4.
52. Khoury, R.; Karam, G. Impact of COVID-19 on Mental Healthcare of Older Adults: Insights from Lebanon (Middle East). *Int. Psychogeriatr.* **2020**, *32*, 1177–1180, doi:10.1017/S104161022000068X.
53. Kim, E.J.; Marrast, L.; Conigliaro, J. COVID-19: Magnifying the Effect of Health Disparities. *J*

GEN INTERN MED **2020**, *35*, 2441–2442, doi:10.1007/s11606-020-05881-4.
54. Koffman, J.; Gross, J.; Etkind, S.N.; Selman, L. Uncertainty and COVID-19: How Are We to Respond? *J R Soc Med* **2020**, *113*, 211–216, doi:10.1177/0141076820930665.
55. Krist, A.H.; DeVoe, J.E.; Cheng, A.; Ehrlich, T.; Jones, S.M. Redesigning Primary Care to Address the COVID-19 Pandemic in the Midst of the Pandemic. *Ann Fam Med* **2020**, *18*, 349–354, doi:10.1370/afm.2557.
56. Krumholz, H.M.; Januzzi, J.L. The American College of Cardiology Roundtable on Research in the Era of COVID-19. *Journal of the American College of Cardiology* **2020**, *76*, 1263–1265, doi:10.1016/j.jacc.2020.08.006.
57. Lam, K.; Lu, A.D.; Shi, Y.; Covinsky, K.E. Assessing Telemedicine Unreadiness Among Older Adults in the United States During the COVID-19 Pandemic. *JAMA Intern Med* **2020**, *180*, 1389, doi:10.1001/jamainternmed.2020.2671.
58. Lee, E.; Loh, W.; Ang, I.; Tan, Y. Plastic Bags as Personal Protective Equipment During the COVID-19 Pandemic: Between the Devil and the Deep Blue Sea. *The Journal of Emergency Medicine* **2020**, *58*, 821–823, doi:10.1016/j.jemermed.2020.04.016.
59. Mahase, E. Covid-19: Charity Cuts Could Put the NHS under Even More Pressure. *BMJ* **2020**, m3261, doi:10.1136/bmj.m3261.
60. Majeed, A.; Molokhia, M. Vaccinating the UK against Covid-19. *BMJ* **2020**, m4654, doi:10.1136/bmj.m4654.
61. Markowitz, J. Virtual Treatment and Social Distancing. *The Lancet Psychiatry* **2020**, *7*, 388–389, doi:10.1016/S2215-0366(20)30140-1.
62. Mateos, R.; Fernández, M.; Franco, M.; Sánchez, M. COVID-19 in Spain. Coming Back to the “New Normality” after 2 Months of Confinement. *Int. Psychogeriatr.* **2020**, *32*, 1169–1172, doi:10.1017/S1041610220001155.
63. Maulik, P.K.; Thornicroft, G.; Saxena, S. Roadmap to Strengthen Global Mental Health Systems to Tackle the Impact of the COVID-19 Pandemic. *Int J Ment Health Syst* **2020**, *14*, 57, doi:10.1186/s13033-020-00393-4.
64. McGarry, B.E.; Grabowski, D.C.; Barnett, M.L. Severe Staffing And Personal Protective Equipment Shortages Faced By Nursing Homes During The COVID-19 Pandemic: Study Examines Staffing and Personal Protective Equipment Shortages Faced by Nursing Homes during the COVID-19 Pandemic. *Health Affairs* **2020**, *39*, 1812–1821, doi:10.1377/hlthaff.2020.01269.
65. Meagher, K.M.; Cummins, N.W.; Bharucha, A.E.; Badley, A.D.; Chlan, L.L.; Wright, R.S. COVID-19 Ethics and Research. *Mayo Clinic Proceedings* **2020**, *95*, 1119–1123, doi:10.1016/j.mayocp.2020.04.019.
66. Medina-Walpole, A. In the COVID-19 Era, Here’s Where We Need Sustained Support. *J Gerontol Nurs* **2020**, *46*, 51–52, doi:10.3928/00989134-20201012-07.
67. Mein, S.A. COVID-19 and Health Disparities: The Reality of “the Great Equalizer.” *J GEN INTERN MED* **2020**, *35*, 2439–2440, doi:10.1007/s11606-020-05880-5.
68. Mills, K.T.; Peacock, E.; Chen, J.; Zimmerman, A.; He, H.; Cyprian, A.; Davis, G.; Fuqua, S.R.; Gilliam, D.S.; Greer, A.; et al. Experiences and Beliefs of Low-Income Patients With Hypertension in Louisiana and Mississippi During the COVID-19 Pandemic. *JAHA* **2021**, *10*, e018510, doi:10.1161/JAHA.120.018510.
69. Miller, A. COVID -19: Not Just an Acute Illness. *Trends Urology & Men Health* **2020**, *11*, 17–19, doi:10.1002/tre.776.
70. Monjur, M.R. COVID-19 and Suicides: The Urban Poor in Bangladesh. *Aust N Z J Psychiatry* **2020**, *54*, 1224–1225, doi:10.1177/0004867420937769.
71. Moynihan, R.; Johansson, M.; Maybee, A.; Lang, E.; Légaré, F. Covid-19: An Opportunity to Reduce Unnecessary Healthcare. *BMJ* **2020**, m2752, doi:10.1136/bmj.m2752.
72. Naser, A.Y.; Dahmash, E.Z.; Al-Rousan, R.; Alwafi, H.; Alrawashdeh, H.M.; Ghoul, I.; Abidine, A.; Bokhary, M.A.; AL-Hadithi, H.T.; Ali, D.; et al. Mental Health Status of the General Population, Healthcare Professionals, and University Students during 2019 Coronavirus Disease Outbreak in Jordan: A Cross-sectional Study. *Brain Behav* **2020**, *10*, doi:10.1002/brb3.1730.

73. Naharci, M.I.; Katipoglu, B.; Tasci, I. Coronavirus 2019 (COVID-19) Outbreak and Geropsychiatric Care for Older Adults: A View from Turkey. *Int. Psychogeriatr.* **2020**, *32*, 1193–1197, doi:10.1017/S1041610220001167.
74. O'Connor, D.; Wilderman, M.; Cao, L.; Cook, K.; Ratnathicam, A.; Simonian, G.; Napolitano, M. Creation of a Dedicated Line Service in the New Jersey Epicenter of COVID-19. *Journal of Vascular Surgery* **2020**, *72*, 1159–1160, doi:10.1016/j.jvs.2020.06.015.
75. O'Reilly-Shah, V.N.; Gentry, K.R.; Van Cleve, W.; Kendale, S.M.; Jabaley, C.S.; Long, D.R. The COVID-19 Pandemic Highlights Shortcomings in US Health Care Informatics Infrastructure: A Call to Action. *Anesthesia & Analgesia* **2020**, *131*, 340–344, doi:10.1213/ANE.0000000000004945.
76. Page, N.; Naik, V.; Singh, P.; Fernandes, P.; Nirabhawane, V.; Chaudhari, S. Homecare and the COVID-19 Pandemic – Experience at an Urban Specialist Cancer Palliative Center. *Indian J Palliat Care* **2020**, *26*, 63, doi:10.4103/IJPC.IJPC_151_20.
77. Pai, R.R.; Alathur, S. Mobile Health Intervention and COVID-19 Pandemic Outbreak: Insights from Indian Context. *IJHG* **2020**, *26*, 42–50, doi:10.1108/IJHG-04-2020-0043.
78. Poussardin, C.; Oulehri, W.; Isner, M.E.; Mertes, P.M.; Collange, O. In-ICU COVID-19 Patients' Characteristics for an Estimation in Post-ICU Rehabilitation Care Requirement. *Anaesthesia Critical Care & Pain Medicine* **2020**, *39*, 479–480, doi:10.1016/j.accpm.2020.06.002.
79. Prior, M.; Delac, K.; Laux, L.; Melone, D. Determining Nursing Education Needs During a Rapidly Changing COVID-19 Environment. *Critical Care Nursing Quarterly* **2020**, *43*, 428–450, doi:10.1097/CNQ.0000000000000328.
80. Sedes, P.R.; Sanz, M.Á.B.; Saera, M.A.B.; RodríguezRey, L.F.C.; Ortega, Á.C.; González, M.C.; López, C. de H.; Santos, E.D.; Barcena, A.E.; Mera, M.J.F.; et al. Contingency Plan for the Intensive Care Services for the COVID-19 Pandemic☆,☆☆. 8.
81. Raffle, A.E.; Pollock, A.M.; Harding-Edgar, L. Covid-19 Mass Testing Programmes. *BMJ* **2020**, m3262, doi:10.1136/bmj.m3262.
82. Rana, U. Elderly Suicides in India: An Emerging Concern during COVID-19 Pandemic. *Int. Psychogeriatr.* **2020**, *32*, 1251–1252, doi:10.1017/S1041610220001052.
83. Ray, K.N.; Ettinger, A.K.; Dwarakanath, N.; Mistry, S.V.; Bey, J.; Chaves-Gnecco, D.; Alston, K.A.; Ripper, L.; Lavage, D.R.; Landsittel, D.P.; et al. Rapid-Cycle Community Assessment of Health-Related Social Needs of Children and Families During Coronavirus Disease 2019. *Academic Pediatrics* **2021**, *21*, 677–683, doi:10.1016/j.acap.2020.10.004.
84. Reger, M.A.; Piccirillo, M.L.; Buchman-Schmitt, J.M. COVID-19, Mental Health, and Suicide Risk Among Health Care Workers: Looking Beyond the Crisis. *J. Clin. Psychiatry* **2020**, *81*, doi:10.4088/JCP.20com13381.
85. Rimmer, A. Covid-19: Most Trainees Have Faced Disruption to Their Training, GMC Survey Shows. *BMJ* **2020**, m4093, doi:10.1136/bmj.m4093.
86. Rokach, A.; Boulazreg, S. The COVID-19 Era: How Therapists Can Diminish Burnout Symptoms through Self-Care. *Curr Psychol* **2020**, doi:10.1007/s12144-020-01149-6.
87. Sandhu, S.; Lemmon, M.E.; Eisenson, H.; Crowder, C.; Bettger, J.P. Addressing the Social Determinants of Health During the COVID-19 Pandemic: Ensuring Equity, Quality, and Sustainability. *Family & Community Health* **2021**, *44*, 78–80, doi:10.1097/FCH.0000000000000290.
88. Schlesinger, T.; Kranke, P.; Zacharowski, K.; Meybohm, P. Coronavirus Threatens Blood Supply: Patient Blood Management Now! *Annals of Surgery* **2020**, *272*, e74, doi:10.1097/SLA.0000000000004086.
89. Scott, I.A. COVID -19 Pandemic and the Tension between the Need to Act and the Need to Know. *Intern Med J* **2020**, *50*, 904–909, doi:10.1111/imj.14929.
90. Sebo, P.; Oertelt-Prigione, S.; de Lucia, S.; Clair, C. COVID-19: A Magnifying Glass for Gender Inequalities in Medical Research. *Br J Gen Pract* **2020**, *70*, 526–527, doi:10.3399/bjgp20X713153.
91. Siegelman, J.N. Reflections of a COVID-19 Long Hauler. *JAMA* **2020**, *324*, 2031,

doi:10.1001/jama.2020.22130.
92. Sienaert, P.; Lambrichts, S.; Popleu, L.; Van Gerven, E.; Buggenhout, S.; Bouckaert, F. Electroconvulsive Therapy During COVID-19-Times: Our Patients Cannot Wait. *The American Journal of Geriatric Psychiatry* **2020**, *28*, 772–775, doi:10.1016/j.jagp.2020.04.013.
93. Sivashanker, K.; Rossman, J.; Resnick, A.; Berwick, D.M. Covid-19 and Decarceration. 2.
94. Skoog, I. COVID-19 and Mental Health among Older People in Sweden. *Int. Psychogeriatr.* **2020**, *32*, 1173–1175, doi:10.1017/S104161022000143X.
95. Spooner, A. Covid-19 in Sweden and UK: Medical Leadership Should Energise Wider Debate. *BMJ* **2020**, m4066, doi:10.1136/bmj.m4066.
96. Stephenson, K.; Sowerby, L.; Hopkins, C.; Kumar, N. The UK National Registry of ENT Surgeons with Coronavirus Disease 2019. *J. Laryngol. Otol.* **2020**, *134*, 665–669, doi:10.1017/S0022215120001747.
97. Suzuki, M.; Hotta, M.; Nagase, A.; Yamamoto, Y.; Hirakawa, N.; Satake, Y.; Nagata, Y.; Suehiro, T.; Kanemoto, H.; Yoshiyama, K.; et al. The Behavioral Pattern of Patients with Frontotemporal Dementia during the COVID-19 Pandemic. *Int. Psychogeriatr.* **2020**, *32*, 1231–1234, doi:10.1017/S104161022000109X.
98. Swain, K. Coping with More than COVID-19. *Lancet Child Adolesc Health* **2020**, *4*, 806, doi:10.1016/S2352-4642(20)30322-9.
99. Vedavanam, K.; Garrett, D.; Davies, N.; Moore, K.J. Old Age Psychiatry Services in the UK Responding to COVID-19. *Int. Psychogeriatr.* **2020**, *32*, 1165–1168, doi:10.1017/S1041610220001015.
100. Veitch, P.; Richardson, K. Nurses Need Support during Covid-19 Pandemic. *J Psychiatr Ment Health Nurs* **2021**, *28*, 303–304, doi:10.1111/jpm.12666.
101. Wand, A.P.F.; Zhong, B.-L.; Chiu, H.F.K.; Draper, B.; De Leo, D. COVID-19: The Implications for Suicide in Older Adults. *Int. Psychogeriatr.* **2020**, *32*, 1225–1230, doi:10.1017/S1041610220000770.
102. Wang, Z. Use the Environment to Prevent and Control COVID-19 in Senior-Living Facilities: An Analysis of the Guidelines Used in China. *Health Environments Research and Design Journal* **2021**, *14*, 130–140, doi:10.1177/1937586720953519.
103. Watterson, A. Covid-19: Inadequate Health and Safety Regulatory Planning in the UK. *BMJ* **2020**, m3174, doi:10.1136/bmj.m3174.
104. Weiss, P.G.; Li, S.-T.T. Leading Change to Address the Needs and Well-Being of Trainees During the COVID-19 Pandemic. *Academic Pediatrics* **2020**, *20*, 735–741, doi:10.1016/j.acap.2020.06.001.
105. Wilson, A.N.; Ravaldi, C.; Scoullar, M.J.L.; Vogel, J.P.; Szabo, R.A.; Fisher, J.R.W.; Homer, C.S.E. Caring for the Carers: Ensuring the Provision of Quality Maternity Care during a Global Pandemic. *Women and Birth* **2021**, *34*, 206–209, doi:10.1016/j.wombi.2020.03.011.
106. Xiang, Y.-T.; Jin, Y.; Cheung, T. Joint International Collaboration to Combat Mental Health Challenges During the Coronavirus Disease 2019 Pandemic. *JAMA Psychiatry* **2020**, *77*, 989, doi:10.1001/jamapsychiatry.2020.1057.
107. Xie, H.; Cheng, X.; Song, X.; Wu, W.; Chen, J.; Xi, Z.; Shou, K. Investigation of the Psychological Disorders in the Healthcare Nurses during a Coronavirus Disease 2019 Outbreak in China. *Medicine* **2020**, *99*, e21662, doi:10.1097/MD.0000000000021662.
108. Zeenny, R.M.; Ramia, E.; Akiki, Y.; Hallit, S.; Salameh, P. Assessing Knowledge, Attitude, Practice, and Preparedness of Hospital Pharmacists in Lebanon towards COVID-19 Pandemic: A Cross-Sectional Study. *J of Pharm Policy and Pract* **2020**, *13*, 54, doi:10.1186/s40545-020-00266-8.
109. Funders' Efforts: Aging And Health, COVID-19: Philanthropic Efforts to Support Older Americans during the COVID-19 Pandemic. *Health Affairs* **2020**, *39*, 1092–1093, doi:10.1377/hlthaff.2020.00599.
