# Supplementary material for: Global Healthcare Needs Related to COVID-19: An Evidence Map of the First Year of the Pandemic
Source: Int J Environ Res Public Health. 2022 Aug 19;19(16):10332. doi: 10.3390/ijerph191610332 (PMC9408445; doi:10.3390/ijerph191610332)
Supplement: Supplementary file 1 [file ijerph-19-10332-s001.zip › S4_DescriptiveSummaryIncludedStudies_17-08-2022.pdf]

Table S4. Descriptive summary of included studies

First author (year)	Type of article	Geographical location	Study aim	Study setting	Participants (target population[a])	Participant details (sample size)	Participants' age (years)<br><br>Participants' gender	Data collection methods	Data collection dates (pandemic outbreak phase details)
Adesoye (2020) [1]	Perspective	Houston, Texas, USA	To develop a tailored approach to training that mitigates impact on resident surgical education and optimizes clinical exposures without compromising safety.	Hospital's surgical department	Healthcare professionals (professional needs)	General surgery residents (sample size unspecified)	Unspecified	Qualitative (online meeting)	Unspecified
Alshmemri (2020) [2]	Original research	Makkah, Saudi Arabia	To explore the perceptions of frontline healthcare workers (nurses) and their opinion about healthcare resources/support while caring for COVID-19 patients. To understand nurses' experiences during the pandemic.	Rural hospital	Healthcare professionals (professional, patient and transversal needs)	Nurses (n = 128)	Age range: 27-57 years<br><br>Women: 62%	Mixed methods: quantitative (questionnaire) and qualitative (interview)	Unspecified
Ashiru-Oredope (2020) [3]	Original research	Multicountry: 31 commonwealth countries in Africa, Asia, America, Europe and the Pacific	To identify and explore the issues facing pharmacy teams across Commonwealth countries during the COVID-19 pandemic.	Pharmacy teams in multiple settings	Healthcare professionals (professional needs)	Pharmacy professionals (n = 545)	Age range: 25-44 years<br><br>Women: 46%	Quantitative (survey)	25/Mar/2020 - 26/Apr/2020
Cao (2020) [4]	Letter to the editor	Beijing, China	To study the basic needs and psychological wellbeing of medical workers in a fever clinic of a Tertiary General Hospital.	Fever clinic in a Tertiary General Hospital	Healthcare professionals (professional needs)	Doctors (n = 16), nurses (n = 19) and clinical technicians (n = 2)	Mean age = 32.8 ± 9.6 SD<br><br>Women:78%	Mixed methods: quantitative (questionnaire) and qualitative (interview)	Unspecified
Cipolotti (2020) [5]	Brief report	London, UK	To evaluate staff distress, listen to their concerns, and obtain information regarding the interventions they deemed most useful. To	Leading tertiary-referral neuroscience specialist hospital	Healthcare professionals (professional and family needs)	Nurses (n = 45), medical doctors (n = 34), allied health	Nurses: mean age = 39.30 ± 11.02; age range = 24-61 years; 71% women	Quantitative (online survey)	24/Apr/2020 - 29/Apr/2020<br><br>(During lockdown, peak

First author (year)	Type of article	Geographical location	Study aim	Study setting	Participants (target population[a])	Participant details (sample size)	Participants' age (years)<br><br>Participants' gender	Data collection methods	Data collection dates (pandemic outbreak phase details)
			understand the needs of staff and rapidly respond accordingly.			professionals (n = 35), non-clinical management and administration staff (n = 29), and other staff (n = 15)	Medical doctors: mean age = 44.88 ± 9.69; age range = 32-65 years; 44% women<br><br>Allied health professionals: mean age = 38.46 ± 9.66 SD; age range = 23-62 years; 63% women<br><br>Non-clinical staff: mean age = 48.38 ± 11.35; age range = 24-67 years, 48% women		of COVID-19 in London)
Digby (2020) [6]	Original research	Melbourne, Australia	To determine the impact of working during the early stage of the COVID-19 pandemic on the well-being of staff.	600-bed acute hospital	Healthcare professionals (professional, patient, family and transversal needs)	Senior medical staff (n = 58), junior medical staff (n = 41), nurses (n = 86), allied health professionals (n = 103), and non-clinical staff (n = 33).	Unspecified	Mixed methods: quantitative and qualitative (survey-based approach)	16/Apr/2020 - 13/May/2020<br><br>(First wave)
Du (2020) [7]	Original research	Hubei province, China	To assess the mental health burden of different professions in China in order to find vulnerable groups, possible influencing factors and successful ways of coping.	Multiple settings	Healthcare professionals (professional needs and family needs)	Doctors (n = 158), nurses (n = 221), other medical staff (n = 24), students (n = 43), teachers/ government staff (n = 60), economy staff (n = 135),	Mean age = 36.92 ± 9.83 SD<br>Age range = 18-71 years<br><br>Women = 72%	Quantitative (survey)	19/Mar/2020 - 07/Apr/2020<br><br>(Final stage of the lockdown in China. Last 4 weeks of the lockdown in Hubei Province)

First author (year)	Type of article	Geographical location	Study aim	Study setting	Participants (target population[a])	Participant details (sample size)	Participants' age (years)<br><br>Participants' gender	Data collection methods	Data collection dates (pandemic outbreak phase details)
						workers/ farmers (n = 26), and "others" (n = 20).			
Elhadi (2020) [8]	Original research	Libya	To determine the prevalence of, and factors associated with, burnout syndrome among Libyan hospital healthcare workers during the COVID-19 pandemic in a time of civil war.	Hospitals	Healthcare professionals (professional and transversal needs)	Internal medicine (n = 223), intensive care (n = 64), emergency medicine (n = 111) and surgical department (n = 134)	Mean age = 33.08 ± 7.25 SD<br><br>Women = 45%	Quantitative (printed and electronic survey)	18/Apr/2020 - 02/May/2020
Feinstein (2020) [9]	Review	Houston, Texas, USA	To address mental health needs of frontline healthcare workers as a result of the COVID-19 pandemic by developing the Healthcare Worker Mental Health COVID-19 Hotline, based on crisis intervention principles.	Multiple settings: Medical School, and hospitals and affiliates	Healthcare professionals (professional, patient, and family needs)	Mental health professionals (n = 43)	Unspecified	Unspecified	Unspecified
Galehdar (2020) [10]	Original research	Khorrambad, Iran	To explore nurses' perception about the care needs of patients with COVID-19.	COVID-19 inpatient wards of General Hospitals	Healthcare professionals (patient, family, and transversal needs)	Nurses (n = 20)	Mean age = 31.95 ± 6.64 SD<br><br>Women = 75%	Qualitative (in-depth semi-structured telephone interviews)	Mar – May, 2020
Glatman-Freedman (2020) [11]	Original research	Israel	To describe the outcomes of a call center created to facilitate communication between health authorities and healthcare providers.	Multiple settings: Hospitals and primary care clinics	Healthcare professionals (professional needs)	Physicians (n = 4135), nurses (n = 1235), emergency service providers (n =	Unspecified	Quantitative (quantitative data on calls received were recorded)	05/Feb/2020 - 14/May/2020<br><br>(Early stages)

First author (year)	Type of article	Geographical location	Study aim	Study setting	Participants (target population[a])	Participant details (sample size)	Participants' age (years)<br><br>Participants' gender	Data collection methods	Data collection dates (pandemic outbreak phase details)
						27), medics (n = 9), administrators (n = 290), general public (n = 162), other (n = 731)			
González-Gil (2020) [12]	Original research	Madrid, Spain	To identify needs related to safety, organization, decision-making, communication and psycho-socio-emotional needs perceived by critical care and emergency nurses during the acute phase of the pandemic.	Public hospitals classified as high, intermediate and low complexity hospitals	Healthcare professionals (professional, patient and family needs)	Nurses (n = 556)	Women = 87%	Quantitative (online questionnaire)	01/Apr/2020 - 15/Apr/2020<br><br>(Peak period of the pandemic in Spain)
Halcomb (2020) [13]	Original research	Australia	To identify Australian primary health care nurses' immediate support needs during the COVID-19 pandemic.	Primary care	Healthcare professionals (professional and transversal needs)	Nurses (n = 637)	Women = 96%	Qualitative (survey)	09/Apr/2020 - 20/Apr/2020<br><br>(Height of the first wave of the pandemic in Australia)
Yu, McIntyre (2020) [14]	Original research	Canada	To determine the impact of and needs from physicians during the early response to the COVID-19 global pandemic, and develop a framework for addressing the pandemic tailored to the needs of Canadian physiatrists.	Multiple settings	Healthcare professionals (professional and patient needs)	Medicine and rehabilitation professionals (n = 136) (including active physiatrists, residents, medical students and non-physiatrists or retired members)	Unspecified	Mixed methods: quantitative and qualitative (survey-based approach)	24/Mar/2020 - 31/Mar/2020<br><br>(Early weeks of the pandemic)

First author (year)	Type of article	Geographical location	Study aim	Study setting	Participants (target population[a])	Participant details (sample size)	Participants' age (years)<br><br>Participants' gender	Data collection methods	Data collection dates (pandemic outbreak phase details)
Lee (2020) [15]	Original research	Multicountry: India and Singapore	To assess the knowledge, attitudes and practices of healthcare workers from India and Singapore on PPE usage and heat stress when performing treatment and care activities during the COVID-19 global pandemic.	Multiple settings	Healthcare professionals (professional needs)	Physicians (n = 66), nurses (n = 61), others (n = 38)	India:<br>Median age = 31 (IQR: 26-38)<br>Women = 42%<br><br>Singapore:<br>Median age = 29 (IQR: 27-33)<br>Women = 64%	Quantitative (printed and electronic survey)	May – June, 2020
Kabir (2020) [16]	Brief report	Stockholm, Sweden	To explore how the pandemic was experienced in the first few months by a nurse who found herself in the midst of the storm.	Elderly residential care	Healthcare professionals (professional, patient, and family needs)	Nurse (n = 1)	Women = 100%	Qualitative (semi-structured interview)	15/May/2020
Kackin (2020) [17]	Original research	Istanbul, Turkey	To determine the experiences and psychosocial problems of nurses caring for patients diagnosed with COVID-19 in Turkey.	Multiple settings	Healthcare professionals (professional and transversal needs)	Nurses (n = 10)	Mean age = 29.7 years<br>Age range = 24-40 years<br><br>Women = 80%	Qualitative (questionnaire and individual in-depth semi-structured interviews)	09/May/2020 - 12/May/2020
Kaplan (2020) [18]	Original research	Philadelphia, Pennsylvania, USA	To query U.S. ICU clinician perspectives on ICU preparedness and concerns regarding delivering COVID-19 patient care.	Hospital (ICU)	Healthcare professionals (professional needs)	Nurses (n = 3470), physicians (n = 664), advanced practice providers (n = 334), respiratory therapists (n = 236), pharmacists (n = 79) and others (n = 82)	Unspecified	Quantitative (online survey)	18/Mar/2020 - 25/Mar/2020<br><br>(Early stages)

First author (year)	Type of article	Geographical location	Study aim	Study setting	Participants (target population[a])	Participant details (sample size)	Participants' age (years)<br><br>Participants' gender	Data collection methods	Data collection dates (pandemic outbreak phase details)
Kerkhoff (2020) [19]	Original research	San Francisco, California, USA	To describe the development of a community-based model ('Test-to Care' Model) and evaluate its reach, feasibility and acceptability.	Community-based	Patients (patient, professional, and transversal needs)	Patients diagnosed with COVID-19 (n = 83)	Median age = 39 years (IQR = 28-50)<br><br>Women = 24%	Mixed methods: Informal interviews and focus groups, brief structured surveys, programmatic data and electronic medical record data	27/Apr/2020 - 14/May/2020
Martin-Delgado (2020) [20]	Original research	Multicountry: Brazil, Colombia and Ecuador	To investigate the needs of healthcare professionals and the technical difficulties faced during the initial outbreak.	Multiple settings	Healthcare professionals (professional and transversal needs)	Physicians (n =534), nurses (n = 263), nursing assistants (n = 171) and other professionals (n = 114)	Unspecified	Mixed methods (online survey)	04/Apr/2020 - 07/May/2020<br><br>(First phase)
Mattila (2020) [21]	Original research	Tampere, Finland	To describe the anxiety levels of Finnish hospital workers during the COVID-19 pandemic and to determine the associations of background variables with hospital workers' anxiety levels.	Specialized medical care centres: a university hospital (tertiary referral center) and a central hospital	Healthcare professionals (professional needs)	Physicians (n = 121, 6%), nurses (n = 1302, 66%) and other hospital staff (e.g, administration, psychologists, logopaedists, chemists; 28%, n = 565)	Largest age group aged 31–40 years (n = 522, 26%).<br><br>Women = 87% (n = 1,731)	Quantitative (online survey)	24/Apr/2020 - 12/May/2020<br><br>(Emergency conditions such as the closure of schools and borders were in force)
Mohindra (2020) [22]	Brief report	North India	To find out the perceived motivations influencing	Multi-specialty tertiary	Healthcare professionals	Unspecified	Unspecified	Qualitative (Interviews)	Unspecified

First author (year)	Type of article	Geographical location	Study aim	Study setting	Participants (target population[a])	Participant details (sample size)	Participants' age (years)<br><br>Participants' gender	Data collection methods	Data collection dates (pandemic outbreak phase details)
			morale amongst healthcare professionals in a multi-specialty tertiary hospital.	hospital	(professional, patient, family and transversal needs)				
Nguyen (2020) [23]	Original research	Idaho, USA	To determine Idaho pharmacists' willingness to provide different COVID-19 related services, assess needed resources to provide such services, and identify and prioritize other unmet community needs.	Pharmacists in multiple settings	Healthcare professionals (professional and transversal needs)	Pharmacists (n = 229)	Unspecified	Mixed methods (online survey)	28/Apr/2020 - 26/May/2020
Ow Yong (2020) [24]	Original research	Singapore	To examine the perception of crisis and emergency risk communication in an acute hospital in response to COVID-19 pandemic in Singapore and to identify its associated enablers and barriers.	Acute care hospital	Healthcare professionals (professional needs)	Physicians (n = 22), nurses (n = 627), allied health professionals (n = 206), administrative staff members (n = 118), ancillary support staff members (n = 180)	Mean age = 37.71 ± 11.46<br><br>Women = 84%	Mixed methods (online survey)	24/Feb/2020 - 28/Feb/2020<br><br>(Initial phase)
Raza (2020) [25]	Original research	Karachi, Pakistan	To explore and understand the factors that impede healthcare professionals to effectively treat COVID-19 patients in Karachi, Pakistan.	Public and private hospitals	Healthcare professionals (professional, patient, family and transversal needs)	Physicians (n = 10), nurses (n = 8)	Mean age = 31.5 years<br><br>Women = 33%	Qualitative (semi-structured in-depth telephone interviews)	06/Apr/2020 - 14/Apr/2020
Redondo-Sama (2020) [26]	Original research	Barcelona and surrounding urban areas,	To analyze the immediate responses in social work to vulnerable groups in the first	Multiple settings and fields of	Healthcare professionals (professional,	Social workers (n = 23)	Largest age group aged less than 45 years (n = 14,	Qualitative (semi-structured	20/Mar/2020 – 27/Mar/2020

First author (year)	Type of article	Geographical location	Study aim	Study setting	Participants (target population[a])	Participant details (sample size)	Participants' age (years)<br><br>Participants' gender	Data collection methods	Data collection dates (pandemic outbreak phase details)
		Spain	15 days of the pandemic in Barcelona and surrounding urban areas in Spain.	intervention including primary care, health, ageing, homelessness, justice, women, disabilities, mental health	service user/patient, family, and transversal needs)		60.87%).<br><br>Women = 83% (n = 19)	interviews)	(First wave: state of alarm and national lockdown declared March 13)
Ripp (2020) [27]	Invited commentary	New York, USA	To describe how a Mount Sinai Health System Employee, Faculty, and Trainee Crisis Support Task force used a rapid needs assessment model to capture the concerns of the workforce related to the COVID-19 pandemic.	Multiple settings: Hospital network and numerous practice sites	Healthcare professionals (professional needs)	Task force representation: Office of Well-being and Resilience (OWBR), Human Resources, Employee Assistance Program, the Departments of Psychiatry, Psychology, Nursing, and Social Work, as well as the recreation office, housing office, security office, infection prevention, institutional leadership, communications team, and department and divisional leadership.	Unspecified	Unspecified (rapid needs assessment)	Mar, 2020

First author (year)	Type of article	Geographical location	Study aim	Study setting	Participants (target population[a])	Participant details (sample size)	Participants' age (years)<br><br>Participants' gender	Data collection methods	Data collection dates (pandemic outbreak phase details)
						(n = unspecified)			
San Juan (2020) [28]	Original research	London, UK	To assess the applicability of well-being guidelines in practice, identify unaddressed healthcare workers' needs and provide recommendations for supporting front-line staff during the current and future pandemics.	Acute care hospitals	Healthcare professionals (professional, patient, family and transversal needs)	Total N = 33 (frontline HCPs)<br><br>Nurses (n = 3), anesthetists (n = 19), other doctors (n = 9), allied health professionals (n = 2)	Women = 61% (n = 20)	Qualitative (semi-structured interviews)	19/Mar/2020 - 24/Apr/2020
Sethi (2020) [29]	Original research	Punjab, Khyber Pakhtunkhwa, and Sindh, Pakistan	To explore COVID-19 impact on health professionals personally and professionally along with the associated challenges.	Multiple settings: Public and private medical and dental institutions	Healthcare professionals (professional and transversal needs)	Total N = 290<br><br>Medicine (n = 237): Basic Sciences (n = 71, 24.48%), Clinical Sciences (n = 166, 57.24%); dentistry (n = 35); medical education (n = 18)	Largest age group aged 36-55 years (n = 190, 65.5%)<br><br>Women = 57% (n = 164)	Qualitative (online survey)	Mar - Apr, 2020
Shanafelt (2020) [30]	Viewpoint (perspective)	California, USA	To explore, during the first week of the COVID-19 pandemic, what health professionals were most concerned about, what messaging and behaviors	Unspecified	Healthcare professionals (professional needs)	Healthcare professionals (n = 69), including physicians, nurses,	Unspecified	Unspecified (listening sessions)	Unspecified<br><br>(First week of the pandemic)

First author (year)	Type of article	Geographical location	Study aim	Study setting	Participants (target population[a])	Participant details (sample size)	Participants' age (years)<br><br>Participants' gender	Data collection methods	Data collection dates (pandemic outbreak phase details)
			they needed from their leaders, and what other tangible sources of support they believed would be most helpful for them.			advanced practice clinicians, residents and fellows			
Simione (2020) [31]	Original research	Italy	To investigate the perception of risk and the worries about COVID-19 infection in both healthcare workers and the general population in Italy, and how this relates to demographic, geographic, and psychological variables such as perceived stress, anxiety, and death anxiety.	Multiple settings	Healthcare professionals (professional and transversal needs)	Total N = 353<br><br>Healthcare professionals (n = 167), including medical doctors, nurses, paramedics and students in medicine/nursing/other medical disciplines; and general population (no-med; n = 186)	Total mean age = 38.26 ± 12.24 yrs<br><br>Total women = 75.07%, n = 265<br><br>HCPs:<br>mean age = 35.56 years,<br>± 9.90 years; women = 133, 80%<br><br>No-med group mean age = 40.69 years ± 13.58 years; women = 132, 71%	Quantitative (online questionnaires)	10/Mar/2020 - 12/Mar/2020<br><br>(Restrictive measures implemented)
Stojanov (2020) [32]	Original research	Nis, Serbia	To evaluate the quality of sleep and health-related quality of life among health care professionals treating patients with COVID-19, quantify the magnitude of symptoms of depression and levels of anxiety, and analyze potential risk factors associated with these symptoms.	Hospitals: Clinical Center, Nis and temporary hospitals	Healthcare professionals (professional needs)	Healthcare professionals (n = 118) including nurses (59.8%) and other professionals	Professionals who treated COVID-19 patients:<br>Mean age = 39.1 ± 7.3<br>Women = 66%<br><br>Professionals who did not treat COVID-19 patients:<br>Mean age = 42.5 ± 9.7<br>Women = 66%	Quantitative (online survey)	Unspecified<br><br>(After 20 days of the establishment of temporary hospitals to combat the COVID-19 pandemic)

First author (year)	Type of article	Geographical location	Study aim	Study setting	Participants (target population[a])	Participant details (sample size)	Participants' age (years)<br><br>Participants' gender	Data collection methods	Data collection dates (pandemic outbreak phase details)
Vanhaecht (2020) [33]	Original research	Flanders, Belgium	To determine the effect of COVID-19 on symptoms of negative and positive mental health and the workforce's experience with various sources of support.	Multiple settings: University and acute hospitals, primary care, residential care centers, and care sites for disabled and mental health care	Healthcare professionals (professional needs)	Healthcare professionals (n = 4509) including paramedics (40.6%, 1831), nurses (33.4%, 1508), doctors (13.4%, 603), management staff (12.2%, 552), and other (0.3%, 15)	Mean age = 41.8 ± 11.4<br><br>Women = 86% (n = 3858)	Quantitative (online survey)	02/Apr/2020-04/May/2020<br><br>(Total lockdown in effect until May 4th)
Yu, Leung (2020) [34]	Original research	Hong Kong	To evaluate the preparedness of family doctors during the early phase of the COVID-19 outbreak in Hong Kong.	Primary Care	Healthcare professionals (professional and transversal needs)	Family doctors (n = 491)	Mean age = 45.0 ± 11.5<br><br>Women = 41%	Mixed methods (online survey)	31/Jan/2020 - 03/Feb/2020<br><br>(COVID-19 had not been announced as a pandemic yet)
Zhang (2020) [35]	Original research	Shanghai and Wuhan, China	To identify stressors and burnout among frontline nurses caring for COVID-19 patients in Wuhan and Shanghai and to explore coping strategies and perceived effective support strategies.	COVID-19-designated hospitals	Healthcare professionals (professional needs)	Nurses (n = 107)	Mean age = 30.28 ± 5.49<br><br>Women = 91%, n = 97/107	Quantitative (online survey)	10/Mar/2020 - 14/Mar/2020<br><br>(Participants had worked on the frontline for more than 1 month)
Wang (2020) [36]	Original research	Shanghai, Zhengzhou, and Kaifeng, China	To identify the environmental factors essential for infection control in senior-living facilities.	Elderly residential care facilities providing multiple levels of care including	Healthcare professionals (professional, resident/ patient, and family needs)	Senior-living facility managers or directors (n = 6)	Mean age = 40<br><br>Women = 67% (n = 4)	Qualitative (phone Interviews)	May 2020<br><br>(Following decreased COVID-19 spread, the represented

First author (year)	Type of article	Geographical location	Study aim	Study setting	Participants (target population[a])	Participant details (sample size)	Participants' age (years)<br><br>Participants' gender	Data collection methods	Data collection dates (pandemic outbreak phase details)
				independent living, assisted living, and nursing care on one site					facilities ended the lockdown on April 7th or 25th and started allowing family members (no other visitors) to visit residents)

[a] Target population refers to the population whose needs were identified relevant to our objectives.

References

1. Adesoye, T.; Davis, C.H.; Del Calvo, H.; Shaikh, A.F.; Chegireddy, V.; Chan, E.Y.; Martinez, S.; Pei, K.Y.; Zheng, F.; Tariq, N. “Optimization of Surgical Resident Safety and Education During the COVID-19 Pandemic – Lessons Learned.” *Journal of Surgical Education* **2021**, *78*, 315–320, doi:10.1016/j.jsurg.2020.06.040.
2. Alshmemri, M.S.; Ramaiah, P. Nurses Experiences and Challenges during COVID 19: Mixed Method Approach. *Journal of Pharmaceutical Research International* **2020**, 81–87, doi:10.9734/jpri/2020/v32i3130920.
3. Ashiru-Oredope, D.; Chan, A.H.Y.; Olaoye, O.; Rutter, V.; Babar, Z.U.D.; Anderson, C.; Anderson, R.; Halai, M.; Matuluko, A.; Nambatya, W.; et al. Needs Assessment and Impact of COVID-19 on Pharmacy Professionals in 31 Commonwealth Countries. *Journal of Pharmaceutical Policy and Practice* **2020**, *13*, 1–11, doi:10.1186/s40545-020-00275-7.
4. Cao, J.; Wei, J.; Zhu, H.; Duan, Y.; Geng, W.; Hong, X.; Jiang, J.; Zhao, X.; Zhu, B. A Study of Basic Needs and Psychological Wellbeing of Medical Workers in the Fever Clinic of a Tertiary General Hospital in Beijing during the COVID-19 Outbreak. *Psychotherapy and Psychosomatics* **2020**, *89*, 252–254, doi:10.1159/000507453.
5. Cipolotti, L.; Chan, E.; Murphy, P.; van Harskamp, N.; Foley, J.A. Factors Contributing to the Distress, Concerns, and Needs of UK Neuroscience Health Care Workers during the COVID-19 Pandemic. *Psychology and Psychotherapy: Theory, Research and Practice* **2021**, *94*, 536–543, doi:10.1111/papt.12298.
6. Digby, R.; Winton-Brown, T.; Finlayson, F.; Dobson, H.; Bucknall, T. Hospital Staff Well-Being during the First Wave of COVID-19: Staff Perspectives. *International Journal of Mental Health Nursing* **2021**, *30*, 440–450, doi:10.1111/inm.12804.
7. Du, J.; Mayer, G.; Hummel, S.; Oetjen, N.; Gronewold, N.; Zafar, A.; Schultz, J.H. Mental Health Burden in Different Professions during the Final Stage of the COVID-19 Lockdown in China: Cross-Sectional Survey Study. *Journal of Medical Internet Research* **2020**, *22*, 1–14, doi:10.2196/24240.
8. Elhadi, M.; Msherghi, A.; Elgzairi, M.; Alhashimi, A.; Bouhuwaish, A.; Biala, M.; Abuelmeda, S.; Khel, S.; Khaled, A.; Alsoufi, A.; et al. Burnout Syndrome Among Hospital Healthcare Workers During the COVID-19 Pandemic and Civil War: A Cross-Sectional Study. *Frontiers in Psychiatry* **2020**, *11*, 1–11, doi:10.3389/fpsyt.2020.579563.
9. Feinstein, R.E.; Kotara, S.; Jones, B.; Shanor, D.; Nemeroff, C.B. A Health Care Workers Mental Health Crisis Line in the Age of COVID-19. *Depression and Anxiety* **2020**, *37*, 822–826, doi:10.1002/da.23073.
10. Galehdar, N.; Toulabi, T.; Kamran, A.; Heydari, H. Exploring Nurses’ Perception about the Care Needs of Patients with COVID-19: A Qualitative Study. *BMC Nursing* **2020**, *19*, 1–8, doi:10.1186/s12912-020-00516-9.
11. Glatman-Freedman, A.; Bromberg, M.; Ram, A.; Lutski, M.; Bassal, R.; Michailevich, O.; Saban, M.; Frankental, D.; Dichtiar, R.; Kruglikov-Moldavsky, A.; et al. A COVID-19 Call Center for Healthcare Providers: Dealing with Rapidly Evolving Health Policy Guidelines. *Israel Journal of Health Policy Research* **2020**, *9*, 1–8, doi:10.1186/s13584-020-00433-x.
12. González-Gil, M.T.; González-Blázquez, C.; Parro-Moreno, A.I.; Pedraz-Marcos, A.; Palmar-Santos, A.; Otero-García, L.; Navarta-Sánchez, M.V.; Alcolea-Cosín, M.T.; Argüello-López, M.T.; Canalejas-Pérez, C.; et al. Nurses’ Perceptions and Demands Regarding COVID-19 Care Delivery in Critical Care Units and Hospital Emergency Services. *Intensive and Critical Care Nursing* **2021**, *62*, 1–9, doi:10.1016/j.iccn.2020.102966.
13. Halcomb, E.; Williams, A.; Ashley, C.; McInnes, S.; Stephen, C.; Calma, K.; James, S. The Support Needs of Australian Primary Health Care Nurses during the COVID-19 Pandemic. *Journal of Nursing Management* **2020**, *28*, 1553–1560, doi:10.1111/jonm.13108.
14. Yu, J.C.; McIntyre, M.; Dow, H.; Robinson, L.; Winston, P. Changes to Rehabilitation Service Delivery and the Associated Physician Perspectives during the COVID-19 Pandemic: A Mixed-Methods Needs Assessment Study. *American Journal of Physical Medicine and Rehabilitation* **2020**, *99*, 775–782, doi:10.1097/PHM.0000000000001516.
15. Lee, J.; Venugopal, V.; Latha, P.K.; Alhadad, S.B.; Leow, C.H.W.; De Goh, N.Y.; Tan, E.; Kjellstrom, T.; Morabito, M.; Lee, J.K.W. Heat Stress and Thermal Perception amongst Healthcare Workers during the Covid-19 Pandemic in India and Singapore. *International Journal of Environmental Research and Public Health* **2020**, *17*, 1–12, doi:10.3390/ijerph17218100.

16. Kabir, Z.N.; Boström, A.M.; Konradsen, H. In Conversation with a Frontline Worker in a Care Home in Sweden during the COVID-19 Pandemic. *Journal of Cross-Cultural Gerontology* **2020**, *35*, 493–500, doi:10.1007/s10823-020-09415-7.
17. Kackin, O.; Ciydem, E.; Aci, O.S.; Kutlu, F.Y. Experiences and Psychosocial Problems of Nurses Caring for Patients Diagnosed with COVID-19 in Turkey: A Qualitative Study. *International Journal of Social Psychiatry* **2021**, *67*, 158–167, doi:10.1177/0020764020942788.
18. Kaplan, L.J.; Kleinpell, R.; Maves, R.C.; Doersam, J.K.; Raman, R.; Ferraro, D.M. Critical Care Clinician Reports on Coronavirus Disease 2019: Results From a National Survey of 4,875 ICU Providers. *Critical Care Explorations* **2020**, *2*, 1–9, doi:10.1097/cce.0000000000000125.
19. Kerkhoff, A.D.; Sachdev, D.; Mizany, S.; Rojas, S.; Gandhi, M.; Peng, J.; Black, D.; Jones, D.; Rojas, S.; Jacobo, J.; et al. Evaluation of a Novel Community-Based COVID-19 “Test-to-Care” Model for Low-Income Populations. *PLoS ONE* **2020**, *15*, 1–18, doi:10.1371/journal.pone.0239400.
20. Martin-Delgado, J.; Viteri, E.; Mula, A.; Serpa, P.; Pacheco, G.; Prada, D.; de Andrade Lourenção, D.C.; Baptista, P.C.P.; Ramirez, G.; Mira, J.J. Availability of Personal Protective Equipment and Diagnostic and Treatment Facilities for Healthcare Workers Involved in COVID-19 Care: A Cross-Sectional Study in Brazil, Colombia, and Ecuador. *PLoS ONE* **2020**, *15*, 1–13, doi:10.1371/journal.pone.0242185.
21. Mattila, E.; Peltokoski, J.; Neva, M.H.; Kaunonen, M.; Helminen, M.; Parkkila, A.K. COVID-19: Anxiety among Hospital Staff and Associated Factors. *Annals of Medicine* **2021**, *53*, 237–246, doi:10.1080/07853890.2020.1862905.
22. Mohindra, R.; R, R.; Suri, V.; Bhalla, A.; Singh, S.M. Issues Relevant to Mental Health Promotion in Frontline Health Care Providers Managing Quarantined/Isolated COVID19 Patients. *Asian Journal of Psychiatry* **2020**, *51*, 1–2, doi:10.1016/j.ajp.2020.102084.
23. Nguyen, E.; Owens, C.T.; Daniels, T.; Boyle, J.; Robinson, R.F. Pharmacists’ Willingness to Provide Coronavirus Disease (COVID-19) Services and the Needs to Support COVID-19 Testing, Management, and Prevention. *Journal of Community Health* **2021**, *46*, 752–757, doi:10.1007/s10900-020-00946-1.
24. Ow Yong, L.M.; Xin, X.; Wee, J.M.L.; Poopalalingam, R.; Kwek, K.Y.C.; Thumboo, J. Perception Survey of Crisis and Emergency Risk Communication in an Acute Hospital in the Management of COVID-19 Pandemic in Singapore. *BMC Public Health* **2020**, *20*, 1–12, doi:10.1186/s12889-020-10047-2.
25. Raza, A.; Matloob, S.; Abdul Rahim, N.F.; Abdul Halim, H.; Khattak, A.; Ahmed, N.H.; Nayab, D.E.; Hakeem, A.; Zubair, M. Factors Impeding Health-Care Professionals to Effectively Treat Coronavirus Disease 2019 Patients in Pakistan: A Qualitative Investigation. *Frontiers in Psychology* **2020**, *11*, 1–11, doi:10.3389/fpsyg.2020.572450.
26. Redondo-Sama, G.; Matulic, V.; Munté-Pascual, A.; Vicente, I. de Social Work during the Covid-19 Crisis: Responding to Urgent Social Needs. *Sustainability (Switzerland)* **2020**, *12*, 1–16, doi:10.3390/su12208595.
27. Ripp, J.; Peccoralo, L.; Charney, D. Attending to the Emotional Well-Being of the Health Care Workforce in a New York City Health System during the COVID-19 Pandemic. *Academic Medicine* **2020**, *95*, 1136–1139, doi:10.1097/ACM.0000000000003414.
28. San Juan, V.N.; Aceituno, D.; Djellouli, N.; Sumray, K.; Regenold, N.; Syversen, A.; Mulcahy Symmons, S.; Dowrick, A.; Mitchinson, L.; Singleton, G.; et al. Mental Health and Well-Being of Healthcare Workers during the COVID-19 Pandemic in the UK: Contrasting Guidelines with Experiences in Practice. *BJPsych Open* **2021**, *7*, 1–9, doi:10.1192/bjo.2020.148.
29. Sethi, B.A.; Sethi, A.; Ali, S.; Aamir, H.S. Impact of Coronavirus Disease (COVID-19) Pandemic on Health Professionals. *Pakistan Journal of Medical Sciences* **2020**, *36*, doi:10.12669/pjms.36.COVID19-S4.2779.
30. Shanafelt, T.; Ripp, J.; Trockel, M. Understanding and Addressing Sources of Anxiety among Health Care Professionals during the COVID-19 Pandemic. *JAMA - Journal of the American Medical Association* **2020**, *323*, 2133–2134, doi:10.1001/jama.2020.5893.
31. Simione, L.; Gnagnarella, C. Differences Between Health Workers and General Population in Risk Perception, Behaviors, and Psychological Distress Related to COVID-19 Spread in Italy. *Frontiers in Psychology* **2020**, *11*, 1–17, doi:10.3389/fpsyg.2020.02166.
32. Stojanov, J.; Malobabic, M.; Stanojevic, G.; Stevic, M.; Milosevic, V.; Stojanov, A. Quality of Sleep and Health-Related Quality of Life among Health Care Professionals Treating Patients with Coronavirus Disease-19. *International Journal of Social Psychiatry* **2021**, *67*, 175–181, doi:10.1177/0020764020942800.

33. Vanhaecht, K.; Seys, D.; Bruyneel, L.; Cox, B.; Kaesemans, G.; Cloet, M.; Van Den Broeck, K.; Cools, O.; De Witte, A.; Lowet, K.; et al. COVID-19 Is Having a Destructive Impact on Health-Care Workers' Mental Well-Being. *International Journal for Quality in Health Care* **2021**, *33*, 1–6, doi:10.1093/intqhc/mzaa158.
34. Yu, E.Y.T.; Leung, W.L.H.; Wong, S.Y.S.; Liu, K.S.N.; Wan, E.Y.F. How Are Family Doctors Serving the Hong Kong Community during the Covid-19 Outbreak? A Survey of Hkcfp Members. *Hong Kong Medical Journal* **2020**, *26*, 176–183, doi:10.12809/hkmj208606.
35. Zhang, Y.; Wang, C.; Pan, W.; Zheng, J.; Gao, J.; Huang, X.; Cai, S.; Zhai, Y.; Latour, J.M.; Zhu, C. Stress, Burnout, and Coping Strategies of Frontline Nurses During the COVID-19 Epidemic in Wuhan and Shanghai, China. *Frontiers in Psychiatry* **2020**, *11*, 1–9, doi:10.3389/fpsyt.2020.565520.
36. Wang, Z. Use the Environment to Prevent and Control COVID-19 in Senior-Living Facilities: An Analysis of the Guidelines Used in China. *Health Environments Research and Design Journal* **2021**, *14*, 130–140, doi:10.1177/1937586720953519.
