# Supplementary material for: Global Healthcare Needs Related to COVID-19: An Evidence Map of the First Year of the Pandemic
Source: Int J Environ Res Public Health. 2022 Aug 19;19(16):10332. doi: 10.3390/ijerph191610332 (PMC9408445; doi:10.3390/ijerph191610332)
Supplement: Supplementary file 1 [file ijerph-19-10332-s001.zip › S5_HCPNeeds_17-08-2022.pdf]

Table S5. Description of the type of needs identified for professionals.

		HEALTHCARE PROFESSIONAL NEEDS		
**Key theme**	**Sub-theme(s)**	**Description**	**No. of studies (references)**	**Illustrative quotes**
BASIC NEEDS (n=16)	**Physical discomfort or distress**	Need to decrease bodily discomfort or distress (e.g, related to personal protective equipment (PPE), exhaustion)	n=11<br><br>Alshmemri [1]<br>Cao [2]<br>Digby [3]<br>Elhadi [4]<br>Feinstein [5]<br>González [6]<br>Kackin [7]<br>Lee [8]<br>San Juan [9]<br>Zhang [10]	*"Of the 37 participants, 6 doctors and 11 nurses reported mild bodily discomfort including tiredness, throat pain, cough, neck and shoulder pain, back pain, headache and nausea, frequent urination, and skin rash. No medical worker was infected with COVID-19." (Cao 254)*
	**Living conditions**	Need to provide lodging support	n=5<br><br>Alshmemri [1]<br>Raza [11]<br>Ripp [12]<br>San Juan [9]<br>Shanafelt [13]	*"Staff [front-line healthcare workers] appreciated the senior management team planning to cover needs beyond HCW [Healthcare workers] clinical work, such as ... optional accommodation … remaining available" (San Juan 6)*
	**Rest**	Need for adequate rest (during shifts and after shifts) to remain physically and mentally healthy and provide high-quality care	n=11<br><br>Alshmemri [1]<br>Cao [2]<br>Digby [3]<br>Feinstein [5]<br>González [6]<br>Halcomb [14]<br>Kabir [15]<br>Lee [8]<br>San Juan [9]<br>Stojanov [16]<br>Zhang [10]	*"Their [medical staff] concentration would decrease after working long hours. Some participants said they 'felt tired and can't have a full sleep' later in the 2- or 3-week period. Many physical and mental challenges of working continuously should be noted, such as the intensity of focus for long periods of time and wearing bulky layers of clothing." (Cao 253)*
	**Diet and hydration**	Need to have access to healthy meals and hydration	n=7<br><br>Alshmemri [1]	*"In addition, 33.6% [emergency nurses] considered it to be the case that the healthcare centre never*

Key theme	Sub-theme(s)		Description	No. of studies (references)	Illustrative quotes
				Cao [2]<br>Digby [3]<br>González [6]<br>Lee [8]<br>Ripp [12]<br>Shanafelt [13]	*provided enough fluid and food to cover the needs caused by physical exhaustion during the work shifts." (González 3)*
		Additional staff	Need to provide sufficient and adequate staffing	n=11<br><br>Cao [2]<br>Cipolotti [17]<br>Digby [3]<br>González [6]<br>Kabir [15]<br>Kackin [7]<br>Kaplan [18]<br>Nguyen [19]<br>Raza [11]<br>Ripp [12]<br>San Juan [9]	*"Almost all respondents to this survey emphasized the importance of additional staff support to cope with the stress and rapid change required of them during the pandemic and beyond." (Digby 7)*
	**Funding and resources (n=17)**	*Facilities and equipment*	Need for adequate facilities, equipment (other than PPE) and resource management to meet changing demands	n=9<br><br>Digby [3]<br>Kackin [7]<br>Kaplan [18]<br>Martin-Delgado [20]<br>Nguyen [19]<br>Ow Yong [21]<br>Raza [11]<br>Ripp [12]<br>Sethi [22]	*"As our hospital systems become overwhelmed by the virus—running well over capacity and facing shortages of critical care medical resources [.]" (Ripp 1)*
		Telehealth	Need for funding and establishing telehealth services	n=3<br><br>Adesoye [23]<br>Halcomb [14]<br>Kaplan [18]	*"One hundred and thirty-four (11.0%) statements were concerned with the funding required to support the provision of quality PHC [primary health care] nursing care. The need for funded nurse-delivered telehealth was the most commonly reported*
OCCUPATIONAL NEEDS (n=34)		*Other funding*			

Key theme	Sub-theme(s)	HEALTHCARE PROFESSIONAL NEEDS Description		No. of studies (references)	Illustrative quotes
					statement regarding funding." (Halcomb 1556)
		Need for other financial support or resources for health services including ability for professionals to provide and bill for services different from the regular services they provide; and overall financial support by health services and public-private donations		n=2<br><br>Feinstein [5]<br>Halcomb [14]	*"Participants identified that in order to provide quality health care to the community, PHC nurses could do more in terms of consultations and assessments, home visits, chronic disease management and psychosocial issues related to COVID-19. However, funding for the provision of these services 'billing for nurses' was identified as an important support required to allow these services to be delivered. Some participants also mentioned the overall need for financial support by health services during COVID-19, including 'funds to keep us open' [.]" (Halcomb 1557)*
	**Coordination (n=8)**	Need to improve coordination activities (e.g., more specified task division, clear chain of command, specifying and deferring tasks).		n=8<br><br>Cao [2]<br>Feinstein [5]<br>Halcomb [14]<br>Kabir [15]<br>Kackin [7]<br>Martin-Delgado [20]<br>Mohindra [24]<br>Shanafelt [13]	*"[F]rontline health care providers managing quarantined/isolated COVID-10 patients … [p]erceived [a] need for a clear chain of command in the management and execution of plans." (Mohindra 1)*
	**Information and communication (n=20)**	*Access to up-to-date information*	Need to receive concise, timely, up-to-date, and clear information about the	n=10<br><br>Ashiru-Oredope [25]<br>Cipolotti [17]<br>Digby [3]<br>Glatman [26]<br>Halcomb [14]<br>Kabir [15]	*"In terms of communication delivery about the pandemic, it was seen as important for 'continuous up to date information' to be provided in a 'consistent and clear' format in a single location to reduce the work in gathering information. Participants spoke of requiring: ´less waffle, more*

Key theme	Sub-theme(s)	Description	No. of studies (references)	Illustrative quotes
		pandemic so that professionals do not have to research everything themselves	Ow Yong [21] Redondo-Sama [27] Ripp [12] Shanafelt [13]	*facts and action plans' and 'daily updates so we don't have to research everything ourselves'." (Halcomb 1555)*
	Workplace communication	Need of improved local communication between colleagues, team members and different services	n=10<br><br>Cipolotti [17] Digby [3] González [6] Halcomb [14] Kabir [15] Kerkhoff [28] Mattila [29] Ow Yong [21] Redondo-Sama [27] San Juan [9]	*"A lack of communication on human resource matters to address staff's welfare issues and concerns promptly was reported [by hospital staff] to have impacted staff morale and perceived support during this crisis." (Ow Yong 9)*
	Communication between health authorities and providers	Need to improve communication between health authorities and providers to better respond to the emergency situation	n=2<br><br>Glatman [26] Yu, McIntyre [30]	*"[Need to] optimize two-way communication between health authorities and providers during a public health emergency:" (Glatman 7)*
	Healthcare professional-patient communication	Need to improve healthcare	n=3<br><br>Alshmemri [1]	*"Telehealth was used as a substitute for most face-to-face clinic appointments. The lack of personal*

Key theme	Sub-theme(s)	Description	No. of studies (references)	Illustrative quotes
		profession al-patient communica tion	Digby [3] Yu, McIntyre [30]	*contact and inability to physically examine patients was raised by some clinicians as a limitation on the accuracy of patient assessment and a barrier to communication. In some cases, clinicians felt that the care they were able to give in this way was suboptimal." (Digby 4)*
	Better communication strategies to keep families informed	Better communica tion between health authorities, healthcare profession als and families to keep families informed	n=3<br><br>Kabir [15]<br>San Juan [9]<br>Yu, McIntyre [30]	*"We [frontline worker] forbade visits at the care homes three weeks before the prime minister announced it. It was very difficult to make the relatives understand. Some tried to break in. Once the prime minister announced it, people began to calm down." (Kabir)*
	Protocolized care	Need for standardiz ed protocols to govern clinical care and logistics planning	n=9<br><br>González [6]<br>Halcomb [14]<br>Kaplan [18]<br>Martín-Delgado [20]<br>Ow Yong [21]<br>San Juan [9]<br>Sethi [22]<br>Yu, Leung [31]<br>Yu, McIntyre [30]	*"More than a quarter of the respondents [hospital staff] who provided qualitative feedback felt that the hospital could provide clearer instructions, instead of just rules, and to have a go-to-person for any clarification on procedures and protocols related to COVID-19." (Ow Yong 8)*
**Recognition and support (n=22)**	*Needs assessment*	Need to listen to healthcare profession als'	n=11<br><br>Cao [2]<br>Cipolotti [17]<br>Digby [3]	*"Leaders should ask team members 'What do you need?' and make every effort to address those needs. Health care professionals do not expect the leader to be able to provide*

Key theme	Sub-theme(s)	HEALTHCARE PROFESSIONAL NEEDS<br>Description	No. of studies (references)	Illustrative quotes
		perspectives to identify and address needs, and involve them in decision-making processes	Feinstein [5]<br>Glatman [26]<br>González [6]<br>Halcomb [14]<br>Kackin [7]<br>Ow Yong [21]<br>Raza [11]<br>Shanafelt [13]	*everything asked for, but having them ask, listen, and acknowledge requests is appreciated. Health care professionals also want to have confidence that their voice and expertise are a part of the conversation as organizations develop their emergency preparedness plans to respond to the pandemic." (Shanafelt 2134)*
	Recognition	Need of recognition of the vital role played	n=8<br>Alshmemri [1]<br>Digby [3]<br>Halcomb [14]<br>Kackin [7]<br>Mohindra [24]<br>Nguyen [19]<br>Ow Yong [21]<br>Shanafelt [13]	*" 'All healthcare workers play a part in day-to-day operations in hospitals regardless of whether patient fronting or not patient fronting. There should be a fair appreciation for all staff' [.]" (Ow Yong 8)*
	Support from leaders	Increased organizational support including support from managers and other leaders within the workplace and within the profession	n=9<br><br>Digby [3]<br>Feinstein [5]<br>Halcomb [14]<br>Kabir [15]<br>Mohindra [24]<br>Ow Yong [21]<br>Sethi [22]<br>Shanafelt [13]<br>Vanhaecht [32]	*"[H]ealth care professionals desire visible leadership during this turbulent time. Leaders, such as hospital executives, nursing leaders, department chairs, and division chiefs, may need to consider innovative ways to be present and connect with their teams given the constraints of social distancing. ... Health care professionals indicate they appreciate leaders visiting hospital units that are caring for patients with COVID-19 regularly to provide reassurance." (Shanafelt 2133, 2134)*
	Community of support	Need to establish a COVID-19	n=3<br><br>Ashiru-Oredope [25]	*"CAPMR [Canadian Association of Physical Medicine and Rehabilitation] should provide avenues for social*

HEALTHCARE PROFESSIONAL NEEDS<br>Key theme	Sub-theme(s)	Description	No. of studies (references)	Illustrative quotes
		community of support	Glatman [26]<br>Yu, McIntyre [30]	*connection and continued wellness during pandemic times. Physiatrists in Canada are spread across a large geographic area, and although those physicians practicing in urban or academic centers may have better connection to colleagues, others practicing in community settings may be more isolated." (Jamie Yu 13)*
	Support advocacy	Support advocacy for physician safety, education, and delivery of care	n=2<br><br>Ashiru-Oredope [25]<br>Yu, McIntyre [30]	*"Please advocate on our [active member of CAMP] behalf as not all hospitals are taking this as seriously for the rehab folks, just for the acute care folks. A lot of us were already underserviced to start." (Jamie Yu 32)*
	Transportation assistance	Need for transportation assistance as work hours and demands increase	n=4<br><br>Martin-Delgado [20]<br>Ripp [12]<br>San Juan [9]<br>Shanafelt [13]	*"Transportation has also become a challenge as public transit and shared rides put health care workers (and the people traveling with them) at risk, but single passenger options are financially unsustainable. " (Ripp 2)*
	Other support	Need for support from the state, the community, and family	n=2<br><br>Mohindra [24]<br>Raza [11]	*" 'Do I [health care professional] need to go on the suicide mission to prove my loyalty as a doctor? What if, all of the HCPs [health care professionals] get infected? Who will be treating the patients? The authorities are hiding their incompetence by blaming the doctors and nurses, which is an offence.' " (Raza 6)*
**Training (n=23)**	*Professional education/training on how*	Need to receive the	n=20	*"Nurses faced challenges in undergoing training simultaneously*

HEALTHCARE PROFESSIONAL NEEDS				
Key theme	Sub-theme(s)	Description	No. of studies (references)	Illustrative quotes
	to face demands of COVID-19	training and support that allows provision of high-quality care to patients during the COVID-19 pandemic while ensuring the health and safety of the staff	Adesoye [23]<br>Alshmemri [1]<br>Ashiru-Oredope [25]<br>Cipolotti [17]<br>Du [33]<br>Glatman [26]<br>González [6]<br>Halcomb [14]<br>Kabir [15]<br>Kaplan [18]<br>Martin-Delgado [20] Mohindra [24]<br>Nguyen [19]<br>Raza [11]<br>Sethi [22]<br>Shanafelt [13]<br>Vanhaecht [32]<br>Yu, Leung [31]<br>Yu, McIntyre [30]<br>Zhang [10]	*while caring for COVID-19 patients as a pandemic requires nurses to acquire more knowledge and skills concerning various dimensions of diagnosis, treatment, and preventing the patients from complications."*<br>*(Alshmeri 86)*
	Redeployment: training and support	Need to receive sufficient training and support in preparation for redeployment and during the onboarding process	n=6<br><br>Digby [3]<br>González [6]<br>Mattila [29]<br>San Juan [9]<br>Shanafelt [13]<br>Yu, McIntyre [30]	*"Fast-track training was used to upskill nurses to work in higher acuity areas in anticipation of an influx of seriously ill patients. Yet in some cases, these upskilled nurses did not feel adequately prepared for new roles. Some clinicians, who usually worked in non-clinical areas, were being redeployed to work in clinical areas despite them feeling that their skills were not current, and they required more education."*<br>*(Digby 4)*
	Specific training and protocols on how to use PPE	Need to receive specific training on	n=10<br><br>Adesoye [23]<br>Digby [3]	*" [Participants reported] [t]he fear of not using PPE properly because of lack of adequate practice (more availability to become comfortable*

Key theme	Sub-theme(s)	Description	No. of studies (references)	Illustrative quotes
		how to use personal protective equipment	González [6]<br>Halcomb [14]<br>Kabir [15]<br>Kaplan [18]<br>Martin-Delgado [20]<br>Mohindra [24]<br>San Juan [9]<br>Yu, Leung [31]	*with the same as prevailing norms)." (Mohindra 1)*
	Training on telehealth/online teaching	Need to receive specific training on telehealth or online teaching	n=4<br><br>Adesoye [23]<br>Ashiru-Oredope [25]<br>Sethi [22]<br>Yu, McIntyre [30]	*"[Issues identified as barriers to provision of virtual care included lack of physical exam capabilities] lack of familiarity with the different technological systems, logistics of scheduling virtual visits, and difficulties with billing and remuneration." (Jamie Yu 9)*
	General learning activities	Need to minimize COVID-19's impact on general learning activities including medical education and research	n=5<br><br>Adesoye [23]<br>Digby [3]<br>San Juan [9]<br>Sethi [22]<br>Yu, McIntyre [30]	*"[M]y [junior medical staff] biggest concern for myself is my training being interrupted, my fellowship examination being postponed and feeling there is zero support or concern from medical workforce around these issues." (Digby 4)*
**Occupational health and safety (n=31)**	*Adequate supply of appropriate PPE*	Need to be able to access an adequate supply of PPE to enable the provision of quality	n=22<br><br>Adesoye [23]<br>Alshmemri [1]<br>Ashiru-Oredope [25]<br>Cao [2]<br>Cipolotti [17]<br>Digby [3]<br>González [6]	*"Personal protective equipment (PPE) was in limited supply at times and there was some confusion about the specific equipment required to be worn in different areas for differing purposes. ... Some non-clinical staff in contact with patients or the public felt that the risk they were exposed to was inadequately assessed and that*

HEALTHCARE PROFESSIONAL NEEDS				
**Key theme**	**Sub-theme(s)**	**Description**	**No. of studies (references)**	**Illustrative quotes**
		routine care and guarantee adequate occupational safety	Halcomb [14]<br>Kabir [15]<br>Kaplan [18]<br>Martin-Delgado [20]<br>Mattila [29]<br>Nguyen [19]<br>Raza [11]<br>Redondo-Sama [27]<br>Ripp [12]<br>San Juan [9]<br>Sethi [22]<br>Shanafelt [13]<br>Wang [34]<br>Yu, Leung [31]<br>Yu, McIntyre[30]	*they should have similar access to PPE as clinicians." (Digby 5)*
	Adequate supply of tests and rapid diagnostic	Need to be able to access an adequate supply of tests and to have a rapid diagnosis for all staff	n=8<br><br>Adesoye [23]<br>Kabir [15]<br>Martin-Delgado [20]<br>Raza [11]<br>San Juan [9]<br>Shanafelt [13]<br>Yu, Leung [31]<br>Yu, McIntyre [30]	*"The lack of diagnostic tests … were prioritized (on a scale of 0–10) by the participants as the main obstacles faced while caring for patients with COVID-19." (Matrín-Delgado 6)*
	Other Information, resources, and procedures to reduce risk of acquiring COVID-19	Need for other up-to-date information, resources, and procedures to reduce the risk of health care	n=24<br><br>Adesoye [23]<br>Alshmemri [1]<br>Cao [2]<br>Cipolotti [17]<br>Digby [3]<br>Du [33]<br>Elhadi [4]<br>González [6]<br>Halcomb [14]	*"Numerous participants [active members of CAMPR] also remarked on concerns regarding personal health and the health of family members, with fear and anxiety of potential infection or spread of infection evident." (Jamie Yu 11)*

HEALTHCARE PROFESSIONAL NEEDS				
**Key theme**	**Sub-theme(s)**	**Description**	**No. of studies (references)**	**Illustrative quotes**
		professionals acquiring the infection and/or being a portal of transmission to close contacts	Kabir [15]<br>Kackin [7]<br>Kaplan [18]<br>Martin-Delgado [20]<br>Mattila [29]<br>Mohindra [24]<br>Ow Yong [21]<br>Raza [11]<br>San Juan [9]<br>Simione [35]<br>Stojanov [16]<br>Shanafelt [13]<br>Yu, Leung [31]<br>Yu, McIntyre [30]<br>Zhang [10]	
**Working conditions (n=24)**	*Job security, fair pay, and leave*	Need for financial stability and support including fair pay and leave and clear human resources policies	n=12<br><br>Alshmemri [1]<br>Digby [3]<br>Elhadi [4]<br>Halcomb [14]<br>Kackin [7]<br>Kaplan [18]<br>Nguyen [19]<br>Ow Yong [21]<br>Sethi [22]<br>Shanafelt [13]<br>Yu, McIntyre [30]<br>Zhang [10]	*"A number of issues were raised regarding threatened employment/ lack of job security and employment conditions (fair pay and leave) that in general impact on the nurses' ability to provide quality care during the pandemic. To provide quality care, the participants felt that they required that 'guaranteed work hours are maintained', including a 'reinstatement' or 'retention' of work hours despite changes in the business models of their workplace due to social distancing requirements." (Halcomb 1557)*
	Reasonable workload	Need for a reasonable workload in consultation with professionals'	n=13<br><br>Alshmemri [1]<br>Cao [2]<br>Cipolotti [17]<br>González [6]<br>Kabir [15]	*"However, participants [front-line healthcare workers] expressed concerns that morale may deteriorate as weeks went by working under strenuous conditions. An important barrier to performing confidently was lack of sleep owing to increased*

HEALTHCARE PROFESSIONAL NEEDS				
**Key theme**	**Sub-theme(s)**	**Description**	**No. of studies (references)**	**Illustrative quotes**
		perspectives	Martin-Delgado [20] Mattila [29] Nguyen [19] Raza [11] Redondo-Sama [27] San Juan [9] Sethi [22] Zhang [10]	*workload to cover staff sickness[.]" (San Juan 6)*
	Work shifts	Need for professionals to work suitable work shifts in consultation with professionals' perspectives	n=10<br><br>Alshmemri [1] Cao [2] Cipolotti [17] Digby [3] Du [33] González [6] Kackin [7] San Juan [9] Shanafelt [13] Zhang [10]	*"The necessarily frequent changes to process and procedure meant that the work environment could be different from one shift to the next. Some staff, especially nurses, found this difficult to cope with." (Digby 6)*
	Remote working	Need to implement high-quality telehealth services and need for organizations and professionals to adjust to the specific conditions of remote working	n=5<br><br>Ashiru-Oredope [25] Digby [3] Kabir [15] Sethi [22] Yu, McIntyre [30]	*"For others with poor internet connection, noisy or distracting housemates (including children), or inadequate physical space and facilities, the home environment was unsuitable and working remotely was difficult." (Digby 4)*
			n=2	

HEALTHCARE PROFESSIONAL NEEDS				
**Key theme**	**Sub-theme(s)**	**Description**	**No. of studies (references)**	**Illustrative quotes**
	Rest space and change rooms	Need to have access to rest space, change rooms and lunchrooms for staff	Digby [3]<br>San Juan [9]	*"Initially, the hospital infrastructure was not equipped with enough space for the change rooms, lunchrooms, and isolation rooms required for the altered conditions." (Digby 5)*
	Appropriate and quality patient care	Need to provide appropriate and quality care during the pandemic	n=8<br><br>Digby [3]<br>Kabir [15]<br>Kackin [7]<br>San Juan [9]<br>Sethi [22]<br>Wang [34]<br>Yu, McIntyre [30]<br>Zhang [10]	*" 'I [nurse] feel that the quality of patient care has dropped down due to the inadequate equipment, uncertain treatment, and the risk of transmission, so I feel sorry'." (Kackin 6)*
PSYCHO-SOCIO-EMOTIONAL NEEDS<br>(n=28)	**Access to mental health professionals (n=10)**	Need to have access to mental health professionals	n=10<br><br>Adesoye [23]<br>Alshmemri [1]<br>Du [33]<br>González [6]<br>Halcomb [14]<br>Kackin [7]<br>Raza [11]<br>San Juan [9]<br>Stojanov [16]<br>Vanhaecht [32]	*"All participants [doctors and nurses] reported that hospitals do not have any interventions or help, which could provide psychological and social assistance to HCPs in COVID-19 pandemic. … 'Literally no one ever thinks of what we are facing in our daily lives. There is no actual channel or helpline for psychologically drained health workers.' " (Raza 7)*
	**Self-care**		n=7	

Key theme	Sub-theme(s)	Description	No. of studies (references)	Illustrative quotes
	**(n=7)**	Need for organizations to encourage self-care and for professionals to engage in self-care in order to enhance well-being	Digby [3]<br>Du [33]<br>González [6]<br>Halcomb [14]<br>Kackin [7]<br>Sethi [22]<br>Yu, McIntyre [30]	*"Achieving adequate self-care was seen to require 'down time with other colleagues', 'hours allocated to staff to maintain staff morale' and a 'longer meal break time, time away from where I am working to debrief myself'." (Halcomb 1557)*
	**Social support (n=20)**	*Childcare and family support*<br>Need for support for personal and family needs as work hours and demands increase and schools and day-care closures occur	n=11<br><br>Cao [2]<br>Digby [3]<br>Kackin [7]<br>Mohindra [24]<br>Ripp [12]<br>San Juan [9]<br>Sethi [22]<br>Shanafelt [13]<br>Simione [35]<br>Yu, Leung [31]<br>Yu, McIntyre [30]	*"Schools were closed, staff were teaching remotely during this period and children were expected to do their schoolwork from home. This was an added burden for parents who were attempting to work at home and supervise children at the same time or were leaving older children at home to study unsupervised." (Digby 4)*
		General social support<br>Need for social support and/or social connection	n=17<br><br>Alshmemri [1]<br>Cao [2]<br>Cipolotti [17]<br>Digby [3]<br>Du [33]<br>Elhadi [4]<br>González [6]<br>Halcomb [14]<br>Kabir [15]<br>Kackin [7]<br>Mohindra [24]<br>Raza [11]	*"Those days that were heaviest, when so many people died, I did not want to go home after work. I could not tell my family what had happened, all the bad things that happened at the care home. ... I talked to a friend who was a priest, about the things that happened. She listened a lot and helped me. I could not relax, could not let go." (Kabir)*

HEALTHCARE PROFESSIONAL NEEDS				
Key theme	Sub-theme(s)	Description	No. of studies (references)	Illustrative quotes
			Ripp [12]<br>San Juan [9]<br>Sethi [22]<br>Yu, McIntyre [30]<br>Zhang [10]	
	**General or other psychological health resources or support (n=25)**	Need to provide other or general psychological health resources or support to cope with stress, anxiety, and other emotional symptoms	n=25<br><br>Adesoye [23]<br>Alshmemri [1]<br>Ashiru-Oredope [25]<br>Cao [2]<br>Cipolotti [17]<br>Digby [3]<br>Du [33]<br>Elhadi [4]<br>Feinstein [5]<br>Kabir [15]<br>Kackin [7]<br>Martin-Delgado [20]<br>Mattila [29]<br>Mohindra [24]<br>Ow Yong [21]<br>Raza [11]<br>Ripp [12]<br>San Juan [9]<br>Sethi [22]<br>Shanafelt [13]<br>Simione [35]<br>Stojanov [16]<br>Vanhaecht [32]<br>Yu, McIntyre [30]<br>Zhang [10]	*"Staff welfare, including for those who were not directly involved in the care of COVID-19 patients should also be looked into [.] …Additionally, they indicated that there would be a need to psychologically support staff to better adhere to social segregation and distancing precautionary measures and this needs to be communicated." (Ow Yong 8)*

References

1. Alshmemri, M.S.; Ramaiah, P. Nurses Experiences and Challenges during COVID 19: Mixed Method Approach. *Journal of Pharmaceutical Research International* **2020**, 81–87, doi:10.9734/jpri/2020/v32i3130920.
2. Cao, J.; Wei, J.; Zhu, H.; Duan, Y.; Geng, W.; Hong, X.; Jiang, J.; Zhao, X.; Zhu, B. A Study of Basic Needs and Psychological Wellbeing of Medical Workers in the Fever Clinic of a Tertiary General Hospital in Beijing during the COVID-19 Outbreak. *Psychotherapy and Psychosomatics* **2020**, *89*, 252–254, doi:10.1159/000507453.
3. Digby, R.; Winton-Brown, T.; Finlayson, F.; Dobson, H.; Bucknall, T. Hospital Staff Well-Being during the First Wave of COVID-19: Staff Perspectives. *International Journal of Mental Health Nursing* **2021**, *30*, 440–450, doi:10.1111/inm.12804.
4. Elhadi, M.; Msherghi, A.; Elgzairi, M.; Alhashimi, A.; Bouhuwaish, A.; Biala, M.; Abuelmeda, S.; Khel, S.; Khaled, A.; Alsoufi, A.; et al. Burnout Syndrome Among Hospital Healthcare Workers During the COVID-19 Pandemic and Civil War: A Cross-Sectional Study. *Frontiers in Psychiatry* **2020**, *11*, 1–11, doi:10.3389/fpsyt.2020.579563.
5. Feinstein, R.E.; Kotara, S.; Jones, B.; Shanor, D.; Nemeroff, C.B. A Health Care Workers Mental Health Crisis Line in the Age of COVID-19. *Depression and Anxiety* **2020**, *37*, 822–826, doi:10.1002/da.23073.
6. González-Gil, M.T.; González-Blázquez, C.; Parro-Moreno, A.I.; Pedraz-Marcos, A.; Palmar-Santos, A.; Otero-García, L.; Navarta-Sánchez, M.V.; Alcolea-Cosín, M.T.; Argüello-López, M.T.; Canalejas-Pérez, C.; et al. Nurses' Perceptions and Demands Regarding COVID-19 Care Delivery in Critical Care Units and Hospital Emergency Services. *Intensive and Critical Care Nursing* **2021**, *62*, 1–9, doi:10.1016/j.iccn.2020.102966.
7. Kackin, O.; Ciydem, E.; Aci, O.S.; Kutlu, F.Y. Experiences and Psychosocial Problems of Nurses Caring for Patients Diagnosed with COVID-19 in Turkey: A Qualitative Study. *International Journal of Social Psychiatry* **2021**, *67*, 158–167, doi:10.1177/0020764020942788.
8. Lee, J.; Venugopal, V.; Latha, P.K.; Alhadad, S.B.; Leow, C.H.W.; De Goh, N.Y.; Tan, E.; Kjellstrom, T.; Morabito, M.; Lee, J.K.W. Heat Stress and Thermal Perception amongst Healthcare Workers during the Covid-19 Pandemic in India and Singapore. *International Journal of Environmental Research and Public Health* **2020**, *17*, 1–12, doi:10.3390/ijerph17218100.
9. San Juan, V.N.; Aceituno, D.; Djellouli, N.; Sumray, K.; Regenold, N.; Syversen, A.; Mulcahy Symmons, S.; Dowrick, A.; Mitchinson, L.; Singleton, G.; et al. Mental Health and Well-Being of Healthcare Workers during the COVID-19 Pandemic in the UK: Contrasting Guidelines with Experiences in Practice. *BJPsych Open* **2021**, *7*, 1–9, doi:10.1192/bjo.2020.148.
10. Zhang, Y.; Wang, C.; Pan, W.; Zheng, J.; Gao, J.; Huang, X.; Cai, S.; Zhai, Y.; Latour, J.M.; Zhu, C. Stress, Burnout, and Coping Strategies of Frontline Nurses During the COVID-19 Epidemic in Wuhan and Shanghai, China. *Frontiers in Psychiatry* **2020**, *11*, 1–9, doi:10.3389/fpsyt.2020.565520.
11. Raza, A.; Matloob, S.; Abdul Rahim, N.F.; Abdul Halim, H.; Khattak, A.; Ahmed, N.H.; Nayab, D.E.; Hakeem, A.; Zubair, M. Factors Impeding Health-Care Professionals to Effectively Treat Coronavirus Disease 2019 Patients in Pakistan: A Qualitative Investigation. *Frontiers in Psychology* **2020**, *11*, 1–11, doi:10.3389/fpsyg.2020.572450.
12. Ripp, J.; Peccoralo, L.; Charney, D. Attending to the Emotional Well-Being of the Health Care Workforce in a New York City Health System during the COVID-19 Pandemic. *Academic Medicine* **2020**, *95*, 1136–1139, doi:10.1097/ACM.0000000000003414.
13. Shanafelt, T.; Ripp, J.; Trockel, M. Understanding and Addressing Sources of Anxiety among Health Care Professionals during the COVID-19 Pandemic. *JAMA - Journal of the American Medical Association* **2020**, *323*, 2133–2134, doi:10.1001/jama.2020.5893.
14. Halcomb, E.; Williams, A.; Ashley, C.; McInnes, S.; Stephen, C.; Calma, K.; James, S. The Support Needs of Australian Primary Health Care Nurses during the COVID-19 Pandemic. *Journal of Nursing Management* **2020**, *28*, 1553–1560, doi:10.1111/jonm.13108.
15. Kabir, Z.N.; Boström, A.M.; Konradsen, H. In Conversation with a Frontline Worker in a Care Home in Sweden during the COVID-19 Pandemic. *Journal of Cross-Cultural Gerontology* **2020**, *35*, 493–500, doi:10.1007/s10823-020-09415-7.
16. Stojanov, J.; Malobabic, M.; Stanojevic, G.; Stevic, M.; Milosevic, V.; Stojanov, A. Quality of Sleep and Health-Related Quality of Life among Health Care Professionals Treating Patients with Coronavirus Disease-19. *International Journal of Social Psychiatry* **2021**, *67*, 175–181, doi:10.1177/0020764020942800.
17. Cipolotti, L.; Chan, E.; Murphy, P.; van Harskamp, N.; Foley, J.A. Factors Contributing to the Distress, Concerns, and Needs of UK Neuroscience Health Care Workers during the COVID-19 Pandemic. *Psychology and Psychotherapy: Theory, Research and Practice* **2021**, *94*, 536–543, doi:10.1111/papt.12298.
18. Kaplan, L.J.; Kleinpell, R.; Maves, R.C.; Doersam, J.K.; Raman, R.; Ferraro, D.M. Critical Care Clinician Reports on Coronavirus Disease 2019: Results From a National Survey of 4,875 ICU Providers. *Critical Care Explorations* **2020**, *2*, 1–9, doi:10.1097/cce.0000000000000125.
19. Nguyen, E.; Owens, C.T.; Daniels, T.; Boyle, J.; Robinson, R.F. Pharmacists' Willingness to Provide Coronavirus Disease (COVID-19) Services and the Needs to Support COVID-19 Testing, Management, and Prevention. *Journal of Community Health* **2021**, *46*, 752–757, doi:10.1007/s10900-020-00946-1.
20. Martin-Delgado, J.; Viteri, E.; Mula, A.; Serpa, P.; Pacheco, G.; Prada, D.; de Andrade Lourenção, D.C.; Baptista, P.C.P.; Ramirez, G.; Mira, J.J. Availability of Personal Protective Equipment and Diagnostic and Treatment Facilities for Healthcare Workers Involved in COVID-19 Care: A Cross-Sectional Study in Brazil, Colombia, and Ecuador. *PLoS ONE* **2020**, *15*, 1–13, doi:10.1371/journal.pone.0242185.
21. Ow Yong, L.M.; Xin, X.; Wee, J.M.L.; Poopalalingam, R.; Kwek, K.Y.C.; Thumboo, J. Perception Survey of Crisis and Emergency Risk Communication in an Acute Hospital in the Management of COVID-19 Pandemic in Singapore. *BMC Public Health* **2020**, *20*, 1–12, doi:10.1186/s12889-020-10047-2.
22. Sethi, B.A.; Sethi, A.; Ali, S.; Aamir, H.S. Impact of Coronavirus Disease (COVID-19) Pandemic on Health Professionals. *Pakistan Journal of Medical Sciences* **2020**, *36*, doi:10.12669/pjms.36.COVID19-S4.2779.
23. Adesoye, T.; Davis, C.H.; Del Calvo, H.; Shaikh, A.F.; Chegireddy, V.; Chan, E.Y.; Martinez, S.; Pei, K.Y.; Zheng, F.; Tariq, N. "Optimization of Surgical Resident Safety and Education During the COVID-19 Pandemic – Lessons Learned." *Journal of Surgical Education* **2021**, *78*, 315–320, doi:10.1016/j.jsurg.2020.06.040.
24. Mohindra, R.; R, R.; Suri, V.; Bhalla, A.; Singh, S.M. Issues Relevant to Mental Health Promotion in Frontline Health Care Providers Managing Quarantined/Isolated COVID19 Patients. *Asian Journal of Psychiatry* **2020**, *51*, 1–2, doi:10.1016/j.ajp.2020.102084.
25. Ashiru-Oredope, D.; Chan, A.H.Y.; Olaoye, O.; Rutter, V.; Babar, Z.U.D.; Anderson, C.; Anderson, R.; Halai, M.; Matuluko, A.; Nambatya, W.; et al. Needs Assessment and Impact of COVID-19 on Pharmacy Professionals in 31 Commonwealth Countries. *Journal of Pharmaceutical Policy and Practice* **2020**, *13*, 1–11, doi:10.1186/s40545-020-00275-7.

26. Glatman-Freedman, A.; Bromberg, M.; Ram, A.; Lutski, M.; Bassal, R.; Michailevich, O.; Saban, M.; Frankental, D.; Dichtiar, R.; Kruglikov-Moldavsky, A.; et al. A COVID-19 Call Center for Healthcare Providers: Dealing with Rapidly Evolving Health Policy Guidelines. *Israel Journal of Health Policy Research* **2020**, *9*, 1–8, doi:10.1186/s13584-020-00433-x.
27. Redondo-Sama, G.; Matulic, V.; Munté-Pascual, A.; Vicente, I. de Social Work during the Covid-19 Crisis: Responding to Urgent Social Needs. *Sustainability (Switzerland)* **2020**, *12*, 1–16, doi:10.3390/su12208595.
28. Kerkhoff, A.D.; Sachdev, D.; Mizany, S.; Rojas, S.; Gandhi, M.; Peng, J.; Black, D.; Jones, D.; Rojas, S.; Jacobo, J.; et al. Evaluation of a Novel Community-Based COVID-19 “Test-to-Care” Model for Low-Income Populations. *PLoS ONE* **2020**, *15*, 1–18, doi:10.1371/journal.pone.0239400.
29. Mattila, E.; Peltokoski, J.; Neva, M.H.; Kaunonen, M.; Helminen, M.; Parkkila, A.K. COVID-19: Anxiety among Hospital Staff and Associated Factors. *Annals of Medicine* **2021**, *53*, 237–246, doi:10.1080/07853890.2020.1862905.
30. Yu, J.C.; McIntyre, M.; Dow, H.; Robinson, L.; Winston, P. Changes to Rehabilitation Service Delivery and the Associated Physician Perspectives during the COVID-19 Pandemic: A Mixed-Methods Needs Assessment Study. *American Journal of Physical Medicine and Rehabilitation* **2020**, *99*, 775–782, doi:10.1097/PHM.0000000000001516.
31. Yu, E.Y.T.; Leung, W.L.H.; Wong, S.Y.S.; Liu, K.S.N.; Wan, E.Y.F. How Are Family Doctors Serving the Hong Kong Community during the Covid-19 Outbreak? A Survey of Hkcfp Members. *Hong Kong Medical Journal* **2020**, *26*, 176–183, doi:10.12809/hkmj208606.
32. Vanhaecht, K.; Seys, D.; Bruyneel, L.; Cox, B.; Kaesemans, G.; Cloet, M.; Van Den Broeck, K.; Cools, O.; De Witte, A.; Lowet, K.; et al. COVID-19 Is Having a Destructive Impact on Health-Care Workers’ Mental Well-Being. *International Journal for Quality in Health Care* **2021**, *33*, 1–6, doi:10.1093/intqhc/mzaa158.
33. Du, J.; Mayer, G.; Hummel, S.; Oetjen, N.; Gronewold, N.; Zafar, A.; Schultz, J.H. Mental Health Burden in Different Professions during the Final Stage of the COVID-19 Lockdown in China: Cross-Sectional Survey Study. *Journal of Medical Internet Research* **2020**, *22*, 1–14, doi:10.2196/24240.
34. Wang, Z. Use the Environment to Prevent and Control COVID-19 in Senior-Living Facilities: An Analysis of the Guidelines Used in China. *Health Environments Research and Design Journal* **2021**, *14*, 130–140, doi:10.1177/1937586720953519.
35. Simione, L.; Gnagnarella, C. Differences Between Health Workers and General Population in Risk Perception, Behaviors, and Psychological Distress Related to COVID-19 Spread in Italy. *Frontiers in Psychology* **2020**, *11*, 1–17, doi:10.3389/fpsyg.2020.02166.
