# Supplementary material for: Global Healthcare Needs Related to COVID-19: An Evidence Map of the First Year of the Pandemic
Source: Int J Environ Res Public Health. 2022 Aug 19;19(16):10332. doi: 10.3390/ijerph191610332 (PMC9408445; doi:10.3390/ijerph191610332)
Supplement: Supplementary file 1 [file ijerph-19-10332-s001.zip › S6_PatientNeeds_17-08-2022.pdf]

Table S6. Description of the type of needs identified for patients

PATIENT NEEDS				
**Key theme**	**Sub-theme(s)**	**Description**	**No. of studies**	**Illustrative quotes**
BASIC NEEDS (n=2)	**Access to essential goods and resources**	Need for support for urgent and regular home deliveries of essential supplies during isolation and need for multilingual and multicultural access to information/services	n=2<br><br>Kerkhoff [1]<br>Redondo-Sama[2]	*"The majority of participants (63%) stated that they would be able to isolate at home, but requested community-based support, including home deliveries [of essential goods e.g., food, personal protective equipment (PPE), cleaning supplies, medication]." (Kerkhoff 8)*
	**Living conditions**	Need to provide lodging support	n=2<br><br>Kerkhoff [1]<br>Redondo-Sama [2]	*"Ten participants said that they would be unable to safely isolate at their current address even with community support and home deliveries (either due to shared living spaces or because they were homeless) and they were provided a temporary room by the SFDPH at an isolation and quarantine hotel." (Kerkhoff 8)*
HEALTHCARE NEEDS (n=11)	**Health insurance**	Need for support obtaining healthcare coverage	n=2<br><br>Kerkhoff [1]<br>Raza [3]	*"[T]he participants expressed deep concerns on the unrealistic charges per day for COVID-19 patient. They expressed that only elites can avail that service." (Raza 6)*
	**Additional healthcare services**	Need to receive additional quality healthcare services	n=3<br><br>Galehdar [4]<br>Kerkhoff [1]<br>Mohindra [5]	*"Patients in isolation have many needs beyond medical management. This includes psychological, social, rehabilitative. All these cannot be looked after by the treating doctor alone." (Mohindra 1)*
	**Appropriate or quality care**	Need to improve the quality of current services to receive appropriate or quality care for all people during the pandemic	n=3<br><br>Galehdar [4]<br>Kabir [6]<br>Redondo-Sama [2]	*"Our physician said in the beginning that all those residents who tested positive would be given palliative care and not curative treatment. My manager and I were very upset by this. This was an absurd situation. Just because a resident tested positive didn't mean she had to die!" (Kabir)*
	**Patient safety**	Need to ensure patient safety by adhering to internationally recognized standard operating procedures and patient safety strategies	n=2<br><br>Galehdar [4]<br>Raza [3]	*"According to the participants, these isolation wards lacked the necessary facilities endangering the lives of patients and HCPs:[Health care professionals]" (Raza 5)*
	**Healthcare professional - patient communication**	Need to improve healthcare professional - patient communication	n=3<br><br>Alshmemri [7]<br>Galehdar [4]<br>Kerkhoff [1]	*"The participants' experiences also showed that the communication of nurses with these patients could reduce their social isolation problems, anxiety, and stress. As mentioned by one of the participants: '…they need a strong connection… ' [.]" (Galehdar 4)*

PATIENT NEEDS				
**Key theme**	**Sub-theme(s)**	**Description**	**No. of studies**	**Illustrative quotes**
	**End-of-life rights**	Need to preserve patient rights during end-of-life care and immediately after death	n=4<br><br>Digby [8]<br>Feinstein [9]<br>Kabir [6]<br>San Juan [10]	*"The transport had so many persons to collect that they needed time and could not come earlier. When they came, we had arranged her so well in clothes that she liked. This is something we do for all. Those who came to take her said 'no, don't do anything. Just leave the patient as it is, you don't need to do anything.' I thought like this……we were near them when they lived, even if we could get infected, but now we were to leave them alone just because she died! It was the routines which stated that the bodies to be put in a plastic bag and marked with the person's identity." (Kabir)*
	**Other**	Need to address other healthcare needs including hospital environment familiarization, need for patient to be fully informed of their health status, access to diagnostic tests for close contacts, and access to up-to-date information on COVID-19 and rehabilitation	n=3<br><br>Galehdar[4]<br>Kerkhoff [1]<br>Yu, McIntyre [11]	*"CAPMR [Canadian Association of Physical Medicine and Rehabilitation] should facilitate collaborative sharing of information and resources for health providers, patients and families. The most frequently noted comments and requests for support involved some degree of sharing information and resources, not only directly related to COVID-19 specific issues, but also for general rehabilitation concerns... [.]" (*Yu, McIntyre *11, 12)*<br><br>*"The analysis of the participants' experiences showed that COVID-19 patients should become familiar with the hospital environment and fully informed of their condition within early hours of arrival to the ward. Since nurses' protective clothes are unfamiliar to the patients, they must become recognizable by writing nurses' names or posting their photos. The data showed that familiarizing patients with the hospital environment and simply explaining the function of medical equipment can encourage them to follow therapeutic instructions." (Gahledar 4)*
PSYCHO-SOCIO-EMOTIONAL NEEDS (n=9)	**Access to mental health professionals**	Need for access to mental health professionals	n=6<br><br>Alshmemri [7]<br>Galehdar [4]<br>González [12]<br>Mohindra [5]<br>Raza [3]<br>Redondo- Sama [2]	*"Qualitative content analysis of the data showed that COVID-19 patients might suffer from many mental disorders and experience a lot of fear and panic during and after the disease. Therefore, they particularly need psychological consulting." (Galehdar 2)*
	**Access to social support (including social welfare and childcare)**	Need for social support services including childcare and social welfare to mitigate economic concerns	n=5<br><br>Galehdar [4]<br>Kerkhoff [1]<br>Mohindra [5]<br>Redondo-Sama [2]<br>Wang [13]	*"Data analysis showed that economic problems were among the main concerns of COVID-19 patients. The participants mentioned that some of the patients were constantly thinking of economic issues, and this would create a great deal of stress, affecting the course of their illness." (Galehdar 5)*

PATIENT NEEDS				
**Key theme**	**Sub-theme(s)**	**Description**	**No. of studies**	**Illustrative quotes**
	**Access to emotional support**	Need to receive emotional support including screening programs to identify patients in need of psychological counselling	n=5<br><br>Galehdar [4]<br>González [12]<br>Kabir [6]<br>Kerkhoff [1]<br>Mohindra [5]	*" '.... I think the first need of ill patients is oxygen, ... but those who have better condition need affection [stated by nurse caring for COVID19 patients]' [.]" (Galehdar 5)*
OTHER SUPPORT NEEDS (n=8)	**Family communication and social interaction**	Need for continuous communication and social interaction with family and friends	n=6<br><br>Galehdar [4]<br>Kabir [6]<br>Raza [3]<br>Redondo-Sama [2]<br>San Juan [10]<br>Wang [13]	*"Based on the data analysis, one of the patients' problems was the lack of familial support. The patients needed to communicate with their families and relatives during isolation and hospitalization. The participants verified that phone or video communications of the patients with their family members created a psychological peace for them and positively affected their recovery process." (Galehdar 5)*
	**Needs assessment**	Need to assess, identify, and address patient needs to provide comprehensive patient care	n=2<br><br>Galehdar [4]<br>Kerkhoff [1]	*"For a comprehensive patient care, their needs should be identified." (Galehdar 5)*
	**Support advocacy**	Support advocacy for services for patients, particularly for vulnerable patients including patients with disabilities and elderly patients	n=2<br><br>Redondo-Sama [2]<br>Yu, McIntyre [11]	*" 'We [active members of CAMPR] need to advocate for our patients with disabilities. I am very concerned about the effect of this COVID-19 situation on their health. For example, all my spasticity clinics are cancelled indefinitely, which may cause a lot of functional problems for the patients.' "* (Yu, McIntyre *12)*
	**Spiritual needs**	Need for spiritual care	n=1<br><br>Galehdar [4]	*"The data analysis showed that one of the patients' needs was to pay attention to their spiritual needs." (Galehdar 5)*

References

1. Kerkhoff, A.D.; Sachdev, D.; Mizany, S.; Rojas, S.; Gandhi, M.; Peng, J.; Black, D.; Jones, D.; Rojas, S.; Jacobo, J.; et al. Evaluation of a Novel Community-Based COVID-19 “Test-to-Care” Model for Low-Income Populations. *PLoS ONE* **2020**, *15*, 1–18, doi:10.1371/journal.pone.0239400.
2. Redondo-Sama, G.; Matulic, V.; Munté-Pascual, A.; Vicente, I. de Social Work during the Covid-19 Crisis: Responding to Urgent Social Needs. *Sustainability (Switzerland)* **2020**, *12*, 1–16, doi:10.3390/su12208595.
3. Raza, A.; Matloob, S.; Abdul Rahim, N.F.; Abdul Halim, H.; Khattak, A.; Ahmed, N.H.; Nayab, D.E.; Hakeem, A.; Zubair, M. Factors Impeding Health-Care Professionals to Effectively Treat Coronavirus Disease 2019 Patients in Pakistan: A Qualitative Investigation. *Frontiers in Psychology* **2020**, *11*, 1–11, doi:10.3389/fpsyg.2020.572450.
4. Galehdar, N.; Toulabi, T.; Kamran, A.; Heydari, H. Exploring Nurses’ Perception about the Care Needs of Patients with COVID-19: A Qualitative Study. *BMC Nursing* **2020**, *19*, 1–8, doi:10.1186/s12912-020-00516-9.
5. Mohindra, R.; R, R.; Suri, V.; Bhalla, A.; Singh, S.M. Issues Relevant to Mental Health Promotion in Frontline Health Care Providers Managing Quarantined/Isolated COVID19 Patients. *Asian Journal of Psychiatry* **2020**, *51*, 1–2, doi:10.1016/j.ajp.2020.102084.
6. Kabir, Z.N.; Boström, A.M.; Konradsen, H. In Conversation with a Frontline Worker in a Care Home in Sweden during the COVID-19 Pandemic. *Journal of Cross-Cultural Gerontology* **2020**, *35*, 493–500, doi:10.1007/s10823-020-09415-7.
7. Alshmemri, M.S.; Ramaiah, P. Nurses Experiences and Challenges during COVID 19: Mixed Method Approach. *Journal of Pharmaceutical Research International* **2020**, 81–87, doi:10.9734/jpri/2020/v32i3130920.
8. Digby, R.; Winton-Brown, T.; Finlayson, F.; Dobson, H.; Bucknall, T. Hospital Staff Well-Being during the First Wave of COVID-19: Staff Perspectives. *International Journal of Mental Health Nursing* **2021**, *30*, 440–450, doi:10.1111/inm.12804.
9. Feinstein, R.E.; Kotara, S.; Jones, B.; Shanor, D.; Nemeroff, C.B. A Health Care Workers Mental Health Crisis Line in the Age of COVID-19. *Depression and Anxiety* **2020**, *37*, 822–826, doi:10.1002/da.23073.
10. San Juan, V.N.; Aceituno, D.; Djellouli, N.; Sumray, K.; Regenold, N.; Syversen, A.; Mulcahy Symmons, S.; Dowrick, A.; Mitchinson, L.; Singleton, G.; et al. Mental Health and Well-Being of Healthcare Workers during the COVID-19 Pandemic in the UK: Contrasting Guidelines with Experiences in Practice. *BJPsych Open* **2021**, *7*, 1–9, doi:10.1192/bjo.2020.148.
11. Yu, J.C.; McIntyre, M.; Dow, H.; Robinson, L.; Winston, P. Changes to Rehabilitation Service Delivery and the Associated Physician Perspectives during the COVID-19 Pandemic: A Mixed-Methods Needs Assessment Study. *American Journal of Physical Medicine and Rehabilitation* **2020**, *99*, 775–782, doi:10.1097/PHM.0000000000001516.
12. González-Gil, M.T.; González-Blázquez, C.; Parro-Moreno, A.I.; Pedraz-Marcos, A.; Palmar-Santos, A.; Otero-García, L.; Navarta-Sánchez, M.V.; Alcolea-Cosín, M.T.; Argüello-López, M.T.; Canalejas-Pérez, C.; et al. Nurses’ Perceptions and Demands Regarding COVID-19 Care Delivery in Critical Care Units and Hospital Emergency Services. *Intensive and Critical Care Nursing* **2021**, *62*, 1–9, doi:10.1016/j.iccn.2020.102966.
13. Wang, Z. Use the Environment to Prevent and Control COVID-19 in Senior-Living Facilities: An Analysis of the Guidelines Used in China. *Health Environments Research and Design Journal* **2021**, *14*, 130–140, doi:10.1177/1937586720953519.
