# Supplementary material for: Global Healthcare Needs Related to COVID-19: An Evidence Map of the First Year of the Pandemic
Source: Int J Environ Res Public Health. 2022 Aug 19;19(16):10332. doi: 10.3390/ijerph191610332 (PMC9408445; doi:10.3390/ijerph191610332)
Supplement: Supplementary file 1 [file ijerph-19-10332-s001.zip › S7_FamilyNeeds_17-08-2022.pdf]

Table S7. Description of the type of needs identified for family members

FAMILY MEMBER NEEDS				
**Key theme**	**Sub-theme(s)**	**Description**	**No. of studies**	**Illustrative quotes**
PSYCHO-SOCIO-EMOTIONAL NEEDS (n=8)	**Access to psycho-emotional support**	Need to provide psychological and/or emotional support to the family members of patients with COVID-19	n=5<br><br>Cipolotti [1]<br>Du [2]<br>Feinstein [3]<br>González [4]<br>Redondo-Sama[5]	*" [A]nd 53.5% considered that it was not possible to meet the psycho-emotional needs of the family members of patients with COVID-19." (Gonzalez 3)*
	**Access to social support (including social welfare and childcare)**	Need to provide social support to the family members of patients with COVID-19, including need for social welfare to mitigate economic concerns and need for childcare support while infected patients are hospitalized	n=3<br><br>González [4]<br>Mohindra [6]<br>Redondo-Sama[5]	*"Social and financial needs (safety and welfare of the family of the patient that may be quarantined) is a major issue that needs addressing." (Mohindra 1)*
	**End-of-life rights**	Need to permit family to accompany dying patients	n=3<br><br>Digby [7]<br>Feinstein [3]<br>San Juan [8]	*"The possibility of patients dying alone generated great distress for HCWs [Health care workers], patients and families. Some HCWs felt that permitting family to accompany dying patients should be a priority." (San Juan 6)*
COMMUNICATION NEEDS (n=7)	**Better communication strategies to keep families informed**	Need for better communication between health authorities, healthcare professionals and families to keep families informed	n=3<br><br>Kabir [9]<br>San Juan [8]<br>Yu, McIntyre [10]	*"We [frontline worker of care home] forbade visits at the care homes three weeks before the prime minister announced it. It was very difficult to make the relatives understand. Some tried to break in. Once the prime minister announced it, people began to calm down." (Kabir)*
	**Communication and social interaction between family and patients/residents**	Needs for continuous communication and interaction between family and patients/residents	n=5<br><br>Galehdar [11]<br>Raza [12]<br>Redondo-Sama[5]<br>San Juan [8]<br>Wang [13]	*"Needs of the residents and families for social interaction were highlighted by all participants [senior-living facility managers in China]." (Wang 6)*<br><br>*"The participants' [nurses caring for COVID-19 patients] experiences indicated that the patients had been having a hard time because of being abandoned by the family, which would lead to separation anxiety." (Galehdar 4)*

References

1. Cipolotti, L.; Chan, E.; Murphy, P.; van Harskamp, N.; Foley, J.A. Factors Contributing to the Distress, Concerns, and Needs of UK Neuroscience Health Care Workers during the COVID-19 Pandemic. *Psychology and Psychotherapy: Theory, Research and Practice* **2021**, *94*, 536–543, doi:10.1111/papt.12298.
2. Du, J.; Mayer, G.; Hummel, S.; Oetjen, N.; Gronewold, N.; Zafar, A.; Schultz, J.H. Mental Health Burden in Different Professions during the Final Stage of the COVID-19 Lockdown in China: Cross-Sectional Survey Study. *Journal of Medical Internet Research* **2020**, *22*, 1–14, doi:10.2196/24240.
3. Feinstein, R.E.; Kotara, S.; Jones, B.; Shanor, D.; Nemeroff, C.B. A Health Care Workers Mental Health Crisis Line in the Age of COVID-19. *Depression and Anxiety* **2020**, *37*, 822–826, doi:10.1002/da.23073.
4. González-Gil, M.T.; González-Blázquez, C.; Parro-Moreno, A.I.; Pedraz-Marcos, A.; Palmar-Santos, A.; Otero-García, L.; Navarta-Sánchez, M.V.; Alcolea-Cosín, M.T.; Argüello-López, M.T.; Canalejas-Pérez, C.; et al. Nurses' Perceptions and Demands Regarding COVID-19 Care Delivery in Critical Care Units and Hospital Emergency Services. *Intensive and Critical Care Nursing* **2021**, *62*, 1–9, doi:10.1016/j.iccn.2020.102966.
5. Redondo-Sama, G.; Matulic, V.; Munté-Pascual, A.; Vicente, I. de Social Work during the Covid-19 Crisis: Responding to Urgent Social Needs. *Sustainability (Switzerland)* **2020**, *12*, 1–16, doi:10.3390/su12208595.
6. Mohindra, R.; R, R.; Suri, V.; Bhalla, A.; Singh, S.M. Issues Relevant to Mental Health Promotion in Frontline Health Care Providers Managing Quarantined/Isolated COVID19 Patients. *Asian Journal of Psychiatry* **2020**, *51*, 1–2, doi:10.1016/j.ajp.2020.102084.
7. Digby, R.; Winton-Brown, T.; Finlayson, F.; Dobson, H.; Bucknall, T. Hospital Staff Well-Being during the First Wave of COVID-19: Staff Perspectives. *International Journal of Mental Health Nursing* **2021**, *30*, 440–450, doi:10.1111/inm.12804.
8. San Juan, V.N.; Aceituno, D.; Djellouli, N.; Sumray, K.; Regenold, N.; Syversen, A.; Mulcahy Symmons, S.; Dowrick, A.; Mitchinson, L.; Singleton, G.; et al. Mental Health and Well-Being of Healthcare Workers during the COVID-19 Pandemic in the UK: Contrasting Guidelines with Experiences in Practice. *BJPsych Open* **2021**, *7*, 1–9, doi:10.1192/bjo.2020.148.
9. Kabir, Z.N.; Boström, A.M.; Konradsen, H. In Conversation with a Frontline Worker in a Care Home in Sweden during the COVID-19 Pandemic. *Journal of Cross-Cultural Gerontology* **2020**, *35*, 493–500, doi:10.1007/s10823-020-09415-7.
10. Yu, J.C.; McIntyre, M.; Dow, H.; Robinson, L.; Winston, P. Changes to Rehabilitation Service Delivery and the Associated Physician Perspectives during the COVID-19 Pandemic: A Mixed-Methods Needs Assessment Study. *American Journal of Physical Medicine and Rehabilitation* **2020**, *99*, 775–782, doi:10.1097/PHM.0000000000001516.
11. Galehdar, N.; Toulabi, T.; Kamran, A.; Heydari, H. Exploring Nurses' Perception about the Care Needs of Patients with COVID-19: A Qualitative Study. *BMC Nursing* **2020**, *19*, 1–8, doi:10.1186/s12912-020-00516-9.
12. Raza, A.; Matloob, S.; Abdul Rahim, N.F.; Abdul Halim, H.; Khattak, A.; Ahmed, N.H.; Nayab, D.E.; Hakeem, A.; Zubair, M. Factors Impeding Health-Care Professionals to Effectively Treat Coronavirus Disease 2019 Patients in Pakistan: A Qualitative Investigation. *Frontiers in Psychology* **2020**, *11*, 1–11, doi:10.3389/fpsyg.2020.572450.
13. Wang, Z. Use the Environment to Prevent and Control COVID-19 in Senior-Living Facilities: An Analysis of the Guidelines Used in China. *Health Environments Research and Design Journal* **2021**, *14*, 130–140, doi:10.1177/1937586720953519.
