# Supplementary material for: Global Healthcare Needs Related to COVID-19: An Evidence Map of the First Year of the Pandemic
Source: Int J Environ Res Public Health. 2022 Aug 19;19(16):10332. doi: 10.3390/ijerph191610332 (PMC9408445; doi:10.3390/ijerph191610332)
Supplement: Supplementary file 1 [file ijerph-19-10332-s001.zip › S8_TransversalNeeds_17-08-2022.pdf]

Table S8. Description of the identified transversal needs

TRANSVERSAL NEEDS				
**Key theme**	**Sub-theme(s)**	**Description**	**No. of studies**	**Illustrative quotes**
PUBLIC SAFETY NEEDS (n=9)	**Adherence to disease prevention protocols**	Need for the general public to follow preventative strategies	n=5<br><br>Alshmemri [1]<br>Galehdar [2]<br>Redondo-Sama [3]<br>Sethi [4]<br>Simione [5]	*"Data analysis indicated that some patients still did not believe in preventive actions and following the disease prevention protocols." (Galehdar 5)*
	**Provision of personal protective equipment (PPE)**	Need for the government and/or health authorities to provide professionals and the general population with PPE to minimize the spread of the virus	n=3<br><br>Halcomb [6]<br>Redondo-Sama [3]<br>Yu, Leung [7]	*" [F]amily doctors in Hong Kong also considered that government and health authorities should be responsible for ensuring steady supply of PPE [personal protective equipment] to frontline healthcare workers and/or the public." (Yu, Leung 181)*
	**More effective public-health policies**	Need for a more effective public health policy to contain the outbreak	n=4<br><br>Digby [8]<br>Martin-Delgado [9]<br>Simione [5]<br>Yu, Leung [7]	*"There was a strong call (n=49, 36%) for more effective public health policy to contain the outbreak, such as border control and/or quarantine measures for returning residents and travellers to reduce imported cases." (Yu, Leung 179)*
INFORMATION AND COMMUNICATION NEEDS (n=16)	**General education and awareness campaigns**	Need to improve general public health-related and COVID-19 education and awareness	n=11<br><br>Alshmemri [1]<br>Galehdar [2]<br>Halcomb [6]<br>Kerkhoff [10]<br>Martin-Delgado [9]<br>Nguyen [11]<br>Raza [12]<br>Redondo-Sama [3]<br>Sethi [4]<br>Simione [5]<br>Yu, Leung [7]	*"Many participants [nurses] also commented that the provision of education to the general public would support them to provide quality care. This included education to promote 'public awareness of risks' and 'continued health promotion to the public not to become complacent' about the spread of COVID-19." (Halcomb 1555, 1556)*
	**Clarifying fake news and misinformation**	Need to spot and combat fake news and control the spread of misinformation	n=6<br><br>Digby [8]<br>Galehdar [2]<br>Halcomb [6]<br>Redondo-Sama [3]<br>Sethi [4]<br>Yu, Leung [7]	*"Various rumours & fake notifications have made conditions worse. One rumour was that government has ordered to kill corona positive patients & their close contacts, so people even with contact history or symptoms were hiding [stated by health professionals]." (Sethi)*

Key theme	Sub-theme(s)	Description	No. of studies	Illustrative quotes
		**TRANSVERSAL NEEDS**		
PUBLIC SAFETY NEEDS (n=9)	**Adherence to disease prevention protocols**	Need for the general public to follow preventative strategies	n=5<br><br>Alshmemri [1]<br>Galehdar [2]<br>Redondo-Sama [3]<br>Sethi [4]<br>Simione [5]	*"Data analysis indicated that some patients still did not believe in preventive actions and following the disease prevention protocols." (Galehdar 5)*
	**Provision of personal protective equipment (PPE)**	Need for the government and/or health authorities to provide professionals and the general population with PPE to minimize the spread of the virus	n=3<br><br>Halcomb [6]<br>Redondo-Sama [3]<br>Yu, Leung [7]	*" [F]amily doctors in Hong Kong also considered that government and health authorities should be responsible for ensuring steady supply of PPE [personal protective equipment] to frontline healthcare workers and/or the public." (Yu, Leung 181)*
	**More effective public-health policies**	Need for a more effective public health policy to contain the outbreak	n=4<br><br>Digby [8]<br>Martin-Delgado [9]<br>Simione [5]<br>Yu, Leung [7]	*"There was a strong call (n=49, 36%) for more effective public health policy to contain the outbreak, such as border control and/or quarantine measures for returning residents and travellers to reduce imported cases." (Yu, Leung 179)*
	**Reducing social stigma**	Need to reduce social stigma associated with COVID-19	n=6<br><br>Elhadi [13]<br>Galehdar [2]<br>Kackin [14]<br>Mohindra [15]<br>Raza [12]<br>San Juan [16]	*"Stigma is an unexplored area. Patient stigma has been found to be a major cause of suppression of travel history and jumping quarantine. ... discharged patients are also likely to face stigma and face difficulty in reintegration with their families and communities. Currently HP [health care provider] is not equipped to handle this issue. This needs to be addressed. … People in the community stigmatise and worry that HP themselves may transmit infection." (Mohindra 1)*
OTHER NEEDS (n=6)	**Support to vulnerable groups**	Need to provide support to vulnerable groups to minimize the spread of the virus	n=1<br><br>Redondo-Sama [3]	*" 'It is of great help for people in situations of vulnerability or who live with people at risk to have hotels that have been enabled for these people [e.g., elderly, homeless, drug consumers; said by social worker].' " (Redondo Sama 11)*
	**Better public-private coordination**	Need for better coordination between the public and private sectors	n=5<br><br>Digby [8]<br>Kerkhoff [10]<br>Mohindra [15]<br>Raza [12]<br>Yu, Leung [7]	*"[S]ocial services, institute and administration should reassure doctors that families needs will be taken care of on priority." (Mohindra 1)*<br><br>*"Despite repeated calls for coordinated care or clear role delineation of family doctors between public and private sectors at times of outbreak, this has still not been achieved."* (Yu, Leung 181)

References

1. Alshmemri, M.S.; Ramaiah, P. Nurses Experiences and Challenges during COVID 19: Mixed Method Approach. *Journal of Pharmaceutical Research International* **2020**, 81–87, doi:10.9734/jpri/2020/v32i3130920.
2. Galehdar, N.; Toulabi, T.; Kamran, A.; Heydari, H. Exploring Nurses' Perception about the Care Needs of Patients with COVID-19: A Qualitative Study. *BMC Nursing* **2020**, *19*, 1–8, doi:10.1186/s12912-020-00516-9.
3. Redondo-Sama, G.; Matulic, V.; Munté-Pascual, A.; Vicente, I. de Social Work during the Covid-19 Crisis: Responding to Urgent Social Needs. *Sustainability (Switzerland)* **2020**, *12*, 1–16, doi:10.3390/su12208595.
4. Sethi, B.A.; Sethi, A.; Ali, S.; Aamir, H.S. Impact of Coronavirus Disease (COVID-19) Pandemic on Health Professionals. *Pakistan Journal of Medical Sciences* **2020**, *36*, doi:10.12669/pjms.36.COVID19-S4.2779.
5. Simione, L.; Gnagnarella, C. Differences Between Health Workers and General Population in Risk Perception, Behaviors, and Psychological Distress Related to COVID-19 Spread in Italy. *Frontiers in Psychology* **2020**, *11*, 1–17, doi:10.3389/fpsyg.2020.02166.
6. Halcomb, E.; Williams, A.; Ashley, C.; McInnes, S.; Stephen, C.; Calma, K.; James, S. The Support Needs of Australian Primary Health Care Nurses during the COVID-19 Pandemic. *Journal of Nursing Management* **2020**, *28*, 1553–1560, doi:10.1111/jonm.13108.
7. Yu, E.Y.T.; Leung, W.L.H.; Wong, S.Y.S.; Liu, K.S.N.; Wan, E.Y.F. How Are Family Doctors Serving the Hong Kong Community during the Covid-19 Outbreak? A Survey of Hkcfp Members. *Hong Kong Medical Journal* **2020**, *26*, 176–183, doi:10.12809/hkmj208606.
8. Digby, R.; Winton-Brown, T.; Finlayson, F.; Dobson, H.; Bucknall, T. Hospital Staff Well-Being during the First Wave of COVID-19: Staff Perspectives. *International Journal of Mental Health Nursing* **2021**, *30*, 440–450, doi:10.1111/inm.12804.
9. Martin-Delgado, J.; Viteri, E.; Mula, A.; Serpa, P.; Pacheco, G.; Prada, D.; de Andrade Lourenção, D.C.; Baptista, P.C.P.; Ramirez, G.; Mira, J.J. Availability of Personal Protective Equipment and Diagnostic and Treatment Facilities for Healthcare Workers Involved in COVID-19 Care: A Cross-Sectional Study in Brazil, Colombia, and Ecuador. *PLoS ONE* **2020**, *15*, 1–13, doi:10.1371/journal.pone.0242185.
10. Kerkhoff, A.D.; Sachdev, D.; Mizany, S.; Rojas, S.; Gandhi, M.; Peng, J.; Black, D.; Jones, D.; Rojas, S.; Jacobo, J.; et al. Evaluation of a Novel Community-Based COVID-19 "Test-to-Care" Model for Low-Income Populations. *PLoS ONE* **2020**, *15*, 1–18, doi:10.1371/journal.pone.0239400.
11. Nguyen, E.; Owens, C.T.; Daniels, T.; Boyle, J.; Robinson, R.F. Pharmacists' Willingness to Provide Coronavirus Disease (COVID-19) Services and the Needs to Support COVID-19 Testing, Management, and Prevention. *Journal of Community Health* **2021**, *46*, 752–757, doi:10.1007/s10900-020-00946-1.
12. Raza, A.; Matloob, S.; Abdul Rahim, N.F.; Abdul Halim, H.; Khattak, A.; Ahmed, N.H.; Nayab, D.E.; Hakeem, A.; Zubair, M. Factors Impeding Health-Care Professionals to Effectively Treat Coronavirus Disease 2019 Patients in Pakistan: A Qualitative Investigation. *Frontiers in Psychology* **2020**, *11*, 1–11, doi:10.3389/fpsyg.2020.572450.
13. Elhadi, M.; Msherghi, A.; Elgzairi, M.; Alhashimi, A.; Bouhuwaish, A.; Biala, M.; Abuelmeda, S.; Khel, S.; Khaled, A.; Alsoufi, A.; et al. Burnout Syndrome Among Hospital Healthcare Workers During the COVID-19 Pandemic and Civil War: A Cross-Sectional Study. *Frontiers in Psychiatry* **2020**, *11*, 1–11, doi:10.3389/fpsyt.2020.579563.
14. Kackin, O.; Ciydem, E.; Aci, O.S.; Kutlu, F.Y. Experiences and Psychosocial Problems of Nurses Caring for Patients Diagnosed with COVID-19 in Turkey: A Qualitative Study. *International Journal of Social Psychiatry* **2021**, *67*, 158–167, doi:10.1177/0020764020942788.
15. Mohindra, R.; R, R.; Suri, V.; Bhalla, A.; Singh, S.M. Issues Relevant to Mental Health Promotion in Frontline Health Care Providers Managing Quarantined/Isolated COVID19 Patients. *Asian Journal of Psychiatry* **2020**, *51*, 1–2, doi:10.1016/j.ajp.2020.102084.
16. San Juan, V.N.; Aceituno, D.; Djellouli, N.; Sumray, K.; Regenold, N.; Syversen, A.; Mulcahy Symmons, S.; Dowrick, A.; Mitchinson, L.; Singleton, G.; et al. Mental Health and Well-Being of Healthcare Workers during the COVID-19 Pandemic in the UK: Contrasting Guidelines with Experiences in Practice. *BJPsych Open* **2021**, *7*, 1–9, doi:10.1192/bjo.2020.148.
